# The relationship between primary colorectal cancer histology and the histopathological growth patterns of corresponding liver metastases

**DOI:** 10.1186/s12885-022-09994-3

**Published:** 2022-08-22

**Authors:** Diederik J. Höppener, Jean-Luc P. L. Stook, Boris Galjart, Pieter M. H. Nierop, Iris D. Nagtegaal, Peter B. Vermeulen, Dirk J. Grünhagen, Cornelis Verhoef, Michail Doukas, PALGA Group

**Affiliations:** 1 Department of Surgical Oncology and Gastrointestinal Surgery, Erasmus MC Cancer Institute, Dr. Molewaterplein 40, 3015 GD Rotterdam, the Netherlands; 2 Department of Pathology, Radboud University Medical Center, Nijmegen, the Netherlands; 3 Translational Cancer Research Unit (GZA Hospitals and University of Antwerp), Antwerp, Belgium; 4 Department of Pathology, Erasmus MC, Rotterdam, the Netherlands

**Keywords:** Histopathological growth patterns, Colorectal cancer, Colorectal liver metastasis, Histopathology, TNM classification, Tumour budding, Desmoplastic reaction, Tumour infiltrating lymphocytes, Crohn’s-like lymphoid reaction

## Abstract

**Background:**

The histopathological growth patterns (HGPs) are a prognostic and predictive biomarker in colorectal cancer liver metastasis (CRLM). This study evaluates the relationship between the HGP and primary colorectal cancer (CRC) histopathology.

**Methods:**

A total of 183 treatment-naive patients with resected CRC and CRLM were included. Thirteen CRC histopathology markers were determined and compared between the desmoplastic and non-desmoplastic HGP; tumour sidedness, pT&pN stage, tumour grade, tumour deposits, perineural- (lympho-)vascular- and extramural venous invasion, peritumoural budding, stroma type, CRC growth pattern, Crohn’s-like lymphoid reaction, and tumour-infiltrating lymphocyte (TIL) density. Logistic regression analysis was performed using both CRC and CRLM characteristics.

**Results:**

Unfavourable CRC histopathology was more frequent in non-desmoplastic CRLM for all markers evaluated, and significantly so for a lower TIL density, *absent* Crohn’s-like lymphoid reaction, and a “non-mature” stroma (all *p* < 0.03). The cumulative prevalence of unfavourable CRC histopathology was significantly higher in patients with non-desmoplastic compared to desmoplastic CRLM, with a median (IQR) of 4 (3–6) vs 2 (1–3.5) unfavourable characteristics observed, respectively (*p* < 0.001). Multivariable regression with 9 CRC histopathology markers and 2 CRLM characteristics achieved good discriminatory performance (AUC = 0.83).

**Conclusions:**

The results of this study associates primary CRC histopathology with the HGP of corresponding liver metastases.

**Supplementary Information:**

The online version contains supplementary material available at 10.1186/s12885-022-09994-3.

## Introduction

The management of colorectal cancer liver metastasis (CRLM) is clinically challenging and requires a multidisciplinary approach. This multidisciplinary need stems from the amenability of CRLM to local therapies such as surgical resection, ablation, and radiotherapy, which is dependent on hepatic tumour load and anatomical location, and the ability of systemic chemotherapy to act upon this through tumour load reduction [1]. Although up to half of all patients can be treated with curative intent, cancer recurrence after surgical treatment of CRLM still occurs in over two-thirds, with long-term cure achieved in approximately one fourth [2–6]. This illustrates a demand for reliable and discriminatory markers to guide clinical decision making, preferably within the pre-treatment setting.

**Fig. 1:**
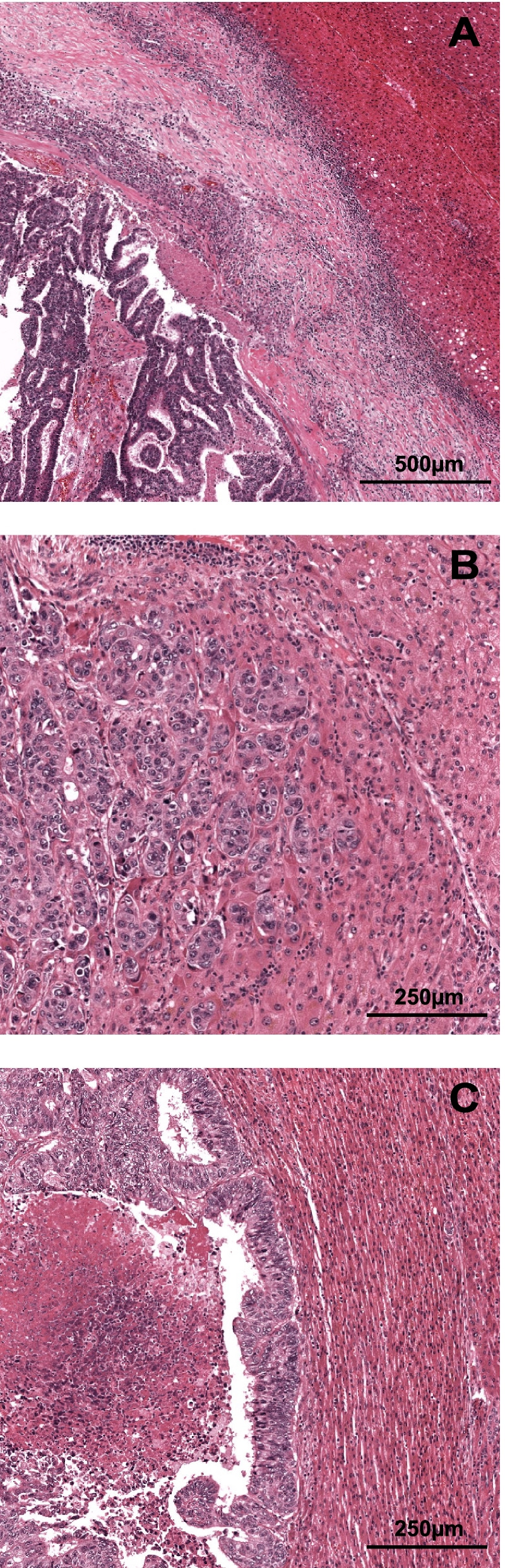
Haematoxylin and Eosin (H&E) stained examples of desmoplastic (**A**) and non-desmoplastic (**B** and **C**) histopathological growth patterns (HGP) of resected colorectal liver metastasis. **A** H&E of the desmoplastic type HGP; note the broad band of desmoplastic stroma separating the tumour from the pre-existing liver parenchyma and the dense lymphocytic infiltrate. **B** H&E of the replacement type HGP; note the infiltration of tumour cells into the pre-existing liver parenchyma with cell to cell contact between tumour cells and hepatocytes all the while retaining some of the liver cell plate architecture. **C** H&E of the rare pushing type HGP; note the well circumscribed margin between the tumour cells and hepatocytes and the compression of the liver cell plates in the pre-existing liver parenchyma

Histopathology studies of CRLM have led to the discovery of distinct histopathological growth patterns (HGP) formed at the interface of liver metastases and the liver parenchyma [7]. A desmoplastic type is recognised in approximately one fifth of resected patients, characterised by the full encapsulation of all liver metastases by desmoplastic stroma (Fig. 1A) [8]. Opposing is the non-desmoplastic type, which is primarily characterised by the complete or partial absence of tumour encapsulation, and secondarily by either invasion (Fig. 1B) or, rarely, compression (Fig. 1C) of the liver parenchyma [8]. The clinical importance of this histopathology marker has been established in multiple cohorts, which reported 5-year overall survival rates of up to 80% for desmoplastic and as low as 40% for non-desmoplastic [8, 9], and have also suggested a benefit for adjuvant systemic chemotherapy for the treatment-naive non-desmoplastic patients only [10]. Since perioperative systemic chemotherapy is considered standard of care in most countries, and current HGP assessment requires a CRLM resection specimen, predicting the HGP beforehand could help identify patients with favourable prognosis that do not require perioperative systemic chemotherapy, and could prevent unnecessary chemotherapy-associated morbidity.

As approximately half of all CRLM are metachronous, resection specimens of primary colorectal cancer are often available [2–4]. A possible clue to assess the HGP preoperatively might therefore lie in the primary CRC histopathology, especially given the number of available and established markers. In addition, associations could reveal underlying biological mechanisms of the distinct HGPs. This study therefore performs an exploratory analysis on the relationship between primary CRC histopathology and the HGP of corresponding CRLM.

## Materials and methods

### Patient selection

A single centre retrospective cohort study was conducted in patients treated surgically with curative intent for CRLM at the Erasmus MC Cancer Institute (Rotterdam, the Netherlands) between January 2000 and February 2019. Eligible patients were those who had had resection of their primary CRC at either the Erasmus MC Cancer Institute or a referring centre affiliated with one of four regional Dutch pathology laboratories (Erasmus MC, Bravis hospital, Maasstad Hospital, or Pathan). Patients who received any preoperative radio- or systemic chemotherapy prior to CRC or CRLM surgery were excluded, as preoperative treatment may alter both CRC histopathology and the HGPs of CRLM [11, 12]. In addition, patients with metachronous CRLM treated with adjuvant chemotherapy had to have had no systemic chemotherapy six months prior to CRLM diagnosis. Data on patient, CRLM, treatment characteristics, and overall survival (OS) was extracted from a prospectively maintained database. Institutional ethical review was obtained from the medical ethics committee of the Erasmus University Medical Center, Rotterdam, the Netherlands (MEC-2018–1743).

### Colorectal liver metastasis HGP

Determination of the liver metastasis HGP was performed previously within the context of retrospective cohort studies [8, 9]. Assessment was at the time performed by at least two trained observers simultaneously on haematoxylin and eosin (H&E) stained tissue sections of resected CRLM, in accordance with international consensus guidelines, and blinded for all patient characteristics (including primary CRC) and survival [7]. In summary, assessment entails the systematic evaluation of the entire tumour liver interface using light microscopy to determine the relative proportion of each of three distinct HGPs (Fig. 1). In line with the upcoming updated consensus guidelines the Rotterdam cut-off was applied and patients were classified as *desmoplastic* if all metastases exclusively displayed a desmoplastic pattern (i.e. 100% desmoplastic, Fig. 1A), and as *non-desmoplastic* otherwise (i.e. < 100% desmoplastic, Fig. 1B and C).

### Primary CRC histopathology

For eligible patients all available H&E slides of resected CRC were requested from the respective pathology laboratories through the nationwide network and registry of histo- and cytopathology in the Netherlands (PALGA) [13]. A literature study was conducted to identify CRC histopathology markers of interest, being those assessable on H&E stained slides of resected CRC, with clinical evidence suggesting a prognostic impact on (overall) survival following CRC resection, and with standardised guidelines and/or detailed methods of assessment. The literature study identified thirteen histopathology markers of interest, which were grouped in four categories. The *classical markers* comprised tumour sidedness, histologic grade, pT-stage, pN-stage, and tumour deposits. Under *invasion markers* were grouped lymphovascular invasion, extramural venous invasion, and perineural invasion. Amongst the *tumour interface markers* were peritumoural budding, CRC growth pattern, and fibrotic stroma type. Lastly, the *immunological markers* consisted of Crohn’s-like lymphoid reaction, and tumour-infiltrating lymphocyte (TIL) density.

A scoring manual was drafted outlining the assessment, definitions, and classifications with corresponding H&E examples for each (novel) individual marker identified (histologic grade, pT&pN-stage, and tumour sidedness were not described). This scoring manual was reviewed by two expert pathologists (PBV and MD) to reach a final consensus (supplementary file 1). A practice session was conducted using 64 digitalised H&E slides of resected CRC from 10 patients to reach agreement on the interpretation and application of the scoring manual. Hereafter the histopathology markers of interest were determined on all available H&E stained slides of included patients. Assessment was performed on a multi-head microscope by a gastro-intestinal pathologist (MD) and several PhD candidates, using the scoring manual as a reference, and blinded for patient characteristics, survival, and liver metastasis HGP. Scoring was done over the course of multiple (> 20) brief morning sessions (1–2 h) to prevent deterioration in assessment quality due to fatigue.

### Classical markers

**Fig. 2:**
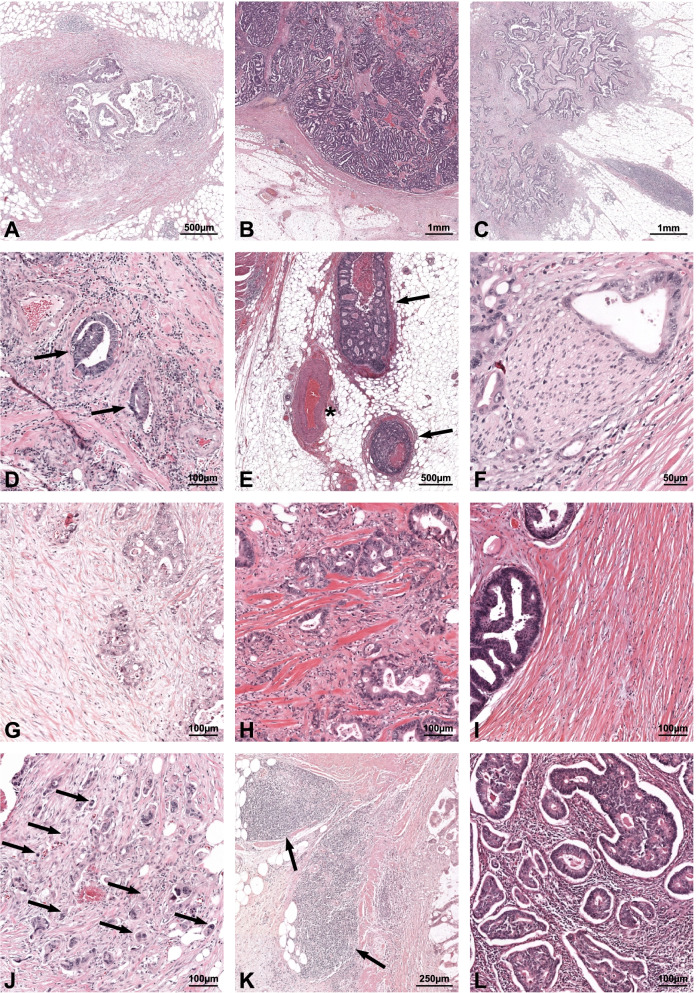
Haematoxylin and Eosin (H&E) stained examples of resected colorectal cancer (CRC) for individual markers. **A** H&E example of a tumour deposit; note the absence of identifiable lymph node tissue and vascular or neural structures. **B** H&E example of the expanding type growth pattern characterized by the pushing/well-circumscribed margin. **C** H&E example of the infiltrating type growth pattern characterized by the diffuse and widespread invasion of normal tissue. **D** H&E example of lymphovascular invasion; the arrows indicate tumour cells located within vascular structures, as can also be identified by the erythrocytes inside both respective lumen. **E** H&E example of extramural venous invasion; the arrows indicate tumour growth into a large vein located in the subserosal fatty tissue and the asterisk indicates the accompanying artery. **F** H&E example of perineural/intraneural invasion of tumour cells inside the nerve sheath. **G** H&E example of the immature fibrotic stroma type characterized by randomly oriented collagen bundles surrounded by myxoid stroma. **H** H&E example of the intermediate fibrotic stroma type characterized by broad bands of brightly eosinophilic hyalinised collaen (ropy-like) intermingled with stroma. **I** H&E example of the mature fibrotic stroma type characterized by multiple fine, mature, and stratiform fibres. **J** H&E example of peritumoural budding; the arrows indicate examples of peritumoural buds located at the invasive margin (not all buds are indicated by arrows). **K** H&E example of Chrohn’s-like lymphoid reaction; the arrows indicate lymphoid aggregates of more than 300 µm in diameter located in the advancing edge of the tumour. **L** H&E example of a high density (50%) of tumour-infiltrating lymphocytes into the intratumoural stromal area at the invasive front

A right-sided tumour was defined as an anatomical CRC localisation proximal to the splenic flexure. The determination of histologic grade, pT&pN-stage, and tumour deposits was done in accordance with the 8^th^ edition of the American Joint Committee on Cancer staging manual for CRC [14]. The 8^th^ edition defines tumour deposits as discrete tumour nodules found within the lymph drainage area of CRC and containing no identifiable lymph node tissue or vascular/neural structures (Fig. 2A).

### Invasion markers

Lymphovascular invasion was defined as the presence of tumour cells within a definite endothelial-lined space (lymphatics or blood vessel) (Fig. 2D) [15], extramural venous invasion as tumour invasion into large veins located in the subserosal or pericolic fat tissue (Fig. 2E) [16], and perineural (or intraneural) invasion as the presence of tumour cells inside the nerve sheath, or when at least one-third of the nerve circumference was encompassed by tumour cells (Fig. 2F) [17].

### Tumour interface markers

Peritumoural budding was assessed in accordance with the 2016 International Tumor Budding Consensus Conference recommendations [18]. Peritumoural buds, defined as a single tumour cell or a cluster of up to four tumour cells without gland formation (Fig. 2J), were counted in a 20 × magnification field at the invasive margin “hotspot” (field with the greatest density of buds in all available slides) and classified using a three-tier system; *Grade I* (low) for 0–4 buds, *Grade II* (intermediate) for 5–9 buds, and *Grade III* (high) for ≥ 10 buds [18]. The CRC growth pattern was assessed according to Jass et al. and classified as either *expanding* or *infiltrative* based on a 50% predominance cut-off [19]. The expanding type is characterised by a pushing or well-circumscribed margin (Fig. 2B), whereas the infiltrative type invades diffusely with widespread penetration of normal tissue (Fig. 2C). The fibrotic stroma type according to Ueno et al. classifies the stroma beyond the muscularis propria (at least pT3 stage) into three distinct types based on morphology; *immature* in case randomly oriented collagen bundles are surrounded by myxoid stroma (Fig. 2G), *intermediate* when broad bands of brightly eosinophilic hyalinised collagen (ropy-like) are intermingled with stroma (Fig. 2H), and *mature* for a stroma composed of multiple fine, mature, and stratiform fibres (Fig. 2I) [20].

### Immunological markers

Crohn’s-Like lymphoid reaction is characterised by lymphoid aggregates of at least 300 µm in diameter observed at the advancing edge of the tumour (Fig. 2K) [21]. Crohn’s-like lymphoid reaction was considered *present* in case at least one aggregate > 300 µm was observed in any slide. Tumour-infiltrating lymphocyte density was assessed by estimating the percentage of mononuclear inflammatory cells over the total intratumoural stromal area at the invasive front (Fig. 2L) [22].

### Statistical analysis

Statistical comparisons between patients with a non-desmoplastic and desmoplastic phenotype were performed to compare baseline patient, CRLM, and treatment characteristics, and to test for associations between individual CRC histopathology markers and the HGP of corresponding CRLM. Nominal variables were compared using the χ^2^ test and are reported as absolute counts with corresponding percentages. Non-parametric ordinal and numerical variables were compared using the Kruskall Wallis test and are reported as medians with corresponding interquartile ranges (IQR). The cumulative prevalence of unfavourable CRC histopathology was compared defined as the number of unfavourable characteristics observed per patient. For markers with more than two classes, a dichotomous classification was adapted, and for TIL density a percentage equal to or below the median was considered unfavourable. Uni- and multivariable binary logistic regression models were fitted with the HGP as dependent variable, and all CRC histopathology and any preoperatively available CRLM characteristics as candidate predictors. The prognostic impact of the HGP on OS following resection of CRLM was estimated by Kaplan–Meier survival analysis. Uni- and multivariable Cox regression analyses were additionally performed on OS following CRLM resection with all CRC histopathology and CRLM characteristics as candidate prognosticators. Given the large number of candidate predictors only those with a univariable *p*-value below 0.2 were entered into the multivariable models. Regression results are reported as multivariable odds ratios (OR) or hazard ratios (HR) with corresponding 95% confidence intervals (CI). Discriminatory capability of the multivariable logistic regression model to predict the HGP was assessed using the Area Under the Curve (AUC) metric of the receiver operating characteristic curve. The statistical significance level was set at a two-sided α of 0.05. All statistical analyses and data visualisation was performed using the R project for statistical computing version 4.1.1 (www.r-project.org), with packages rms (6.0–1), tableone (0.12.0), pROC (1.16.2), circlize (0.4.11) [23], and ggplot2 (3.3.2).

## Results

Primary CRC slides were requested for a total of 196 eligible patients through PALGA and were available for 183 (93%). A desmoplastic HGP was observed in 31 (17%) out of the 183 patients included for analysis. Baseline patient, CRLM, and treatment characteristics stratified by HGP are reported in Table 1. Patients with a non-desmoplastic HGP had a significantly larger CRLM diameter (median [IQR]: 3.2 [2.2, 4.1] vs 2.0 [1.3, 3.0] cm, *p* < 0.001), a significantly higher preoperative serum carcinoembryonic antigen level (median [IQR]: 12.0 [5.0, 44.7] vs 5.7 [3.2, 10.6] µg/L, *p* = 0.002), and more often had positive surgical margins upon CRLM resection (*n* = 14 [10%] vs *n* = 0 [0%], *p* = 0.07) (Table 1). Within the 119 patients with metachronous CRLM non-desmoplastic patients more often received adjuvant systemic chemotherapy (*n* = 46 [45%] vs *n* = 3 [19%], *p* = 0.05, Table 1).

**Table 1:** Baseline CRLM characteristics stratified by histopathological growth pattern

			Non-desmoplastic	Desmoplastic	
		*missing (%)*	*n* = 152 (%)	*n* = 31 (%)	*p*-value
Age at resection CRLM—*(median [IQR])*			66.5 [59.0, 74.0]	68.0 [62.0, 76.5]	0.26
Gender	*Male*		55 (36)	9 (29)	0.45
	*Female*		97 (64)	22 (71)	
Resection timing	*Liver first*		1 (1)	0 (0)	0.07
	*Primary first*		141 (93)	25 (81)	
	*Synchronous*		10 (7)	6 (19)	
Metastasis timing^a^	*Metachronous*		103 (68)	16 (52)	0.09
	*Synchronous*		49 (32)	15 (48)	
Disease-free interval in months^a^—*(median [IQR])*			12.0 [0.0, 24.2]	4.0 [0.0, 20.5]	0.11
Adjuvant CTx following CRC resection^b^	*No*		57 (55)	13 (81)	0.05
	*Yes*		46 (45)	3 (19)	
CRLM distribution	*Unilobar*		127 (84)	28 (90)	0.34
	*Bilobar*		25 (16)	3 (10)	
Number of CRLM—*(median [IQR])*		*1 (1)*	1.0 [1.0, 2.0]	1.0 [1.0, 2.0]	0.50
Diameter of largest CRLM in cm—*(median [IQR])*		*1 (1)*	3.2 [2.2, 4.1]	2.0 [1.3, 3.0]	< 0.001
Preoperative CEA in µg/L—*(median [IQR])*		*13 (7)*	12.0 [5.0, 44.7]	5.7 [3.2, 10.6]	0.002
Concomitant ablation	*No*		134 (88)	27 (87)	0.66
	*RFA*		15 (10)	4 (13)	
	*MWA*		3 (2)	0 (0)	
Resection margin involved	*No*	*4 (2)*	134 (91)	31 (100)	0.07
	*Yes*		14 (9)	0 (0)	
Extrahepatic disease	*No*		136 (89)	30 (97)	0.20
	*Yes*		16 (11)	1 (3)	

*CEA* carcinoembryonic antigen, *CRLM* colorectal liver metastasis, *CTx* chemotherapy, *IQR* interquartile range, *MWA* microwave ablation, *RFA* radiofrequency ablation

^a^Between resection of primary tumour and detection of CRLM. Synchronous is defined as CRLM diagnosed prior to or within 3 months following CRC resection

^b^Within the 119 patients with metachronous CRLM

### Primary CRC histopathology

**Fig. 3:**
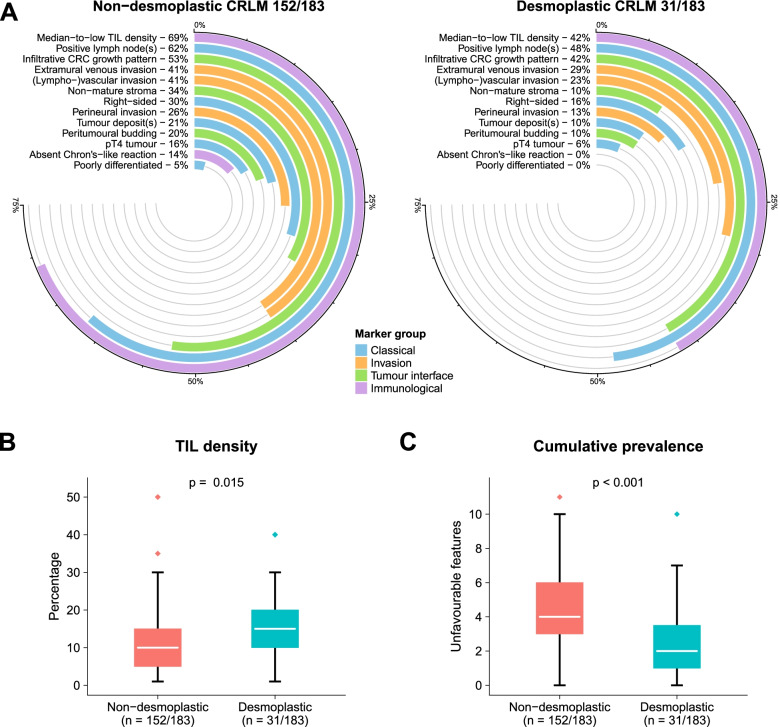
**A** Graphical representation of the frequency of individual unfavourable histopathology features observed in the primary colorectal cancers (CRC) of patients with corresponding non-desmoplastic (left) and desmoplastic (right) liver metastases. **B** and **C** Boxplots demonstrating the distribution of tumour-infiltrating lymphocyte (TIL) density (**B**) and the cumulative prevalence of unfavourable CRC histopathology characteristics (**C**) in patients with corresponding non-desmoplastic (red) and desmoplastic (blue) liver metastases. The *p*-value represents the result of the non-parametric Kruskall Wallis test

A total of 913 H&E slides of resected CRC were reviewed. The median number of tumour containing slides assessed per patient was 4 (IQR: 3–6) and did not differ between patients with corresponding non-desmoplastic (4 IQR [3-7]) and desmoplastic (4 IQR [3–5.5]) CRLM (*p* = 0.27). The great majority were adenocarcinomas (*n* = 179, 98%), with only 4 (2%) mucinous adenocarcinomas, which were equally distributed between non-desmoplastic (*n* = 3, 2%) and desmoplastic (*n* = 1, 3%) patients (*p* = 0.66). Comparisons of all primary CRC histopathology markers stratified by corresponding liver metastasis HGP is reported in Table 2 and Fig. 3A-C.

**Table 2:** Primary CRC hisopathology compared for liver metastasis histopathological growth pattern

			Non-desmoplastic	Desmoplastic	
		*missing (%)*	*n* = 152 (%)	*n* = 31 (%)	*p*-value
*Classical markers*					
Primary tumour location	*Rectum*		17 (11)	3 (10)	0.23
	*Left-sided*		89 (59)	23 (74)	
	*Right-sided*		46 (30)	5 (16)	
Right-sided tumour	*No*		106 (70)	26 (84)	0.11
	*Yes*		46 (30)	5 (16)	
Differentiation grade	*Well / moderate (G1/G2)*		145 (95)	31 (100)	0.22
	*Poor (G3)*		7 (5)	0 (0)	
pT-stage	*pT1*		3 (2)	0 (0)	0.53
	*pT2*		15 (10)	3 (10)	
	*pT3*		109 (72)	26 (84)	
	*pT4a*		19 (12)	1 (3)	
	*pT4b*		6 (4)	1 (3)	
pT4-stage	*No*		127 (84)	29 (94)	0.15
	*Yes*		25 (16)	2 (6)	
pN-stage	*N0*		58 (38)	16 (52)	0.52
	*N1a*		28 (18)	6 (19)	
	*N1b*		30 (20)	3 (10)	
	*N1c*		4 (3)	0 (0)	
	*N2a*		15 (10)	4 (13)	
	*N2b*		17 (11)	2 (6)	
Positive lymph nodes	*No*		58 (38)	16 (52)	0.16
	*Yes*		94 (62)	15 (48)	
Tumour deposits	*No*		120 (79)	28 (90)	0.14
	*Yes*		32 (21)	3 (10)	
*Invasion markers*					
(lympho-)vascular invasion	*No*		90 (59)	24 (77)	0.06
	*Yes*		62 (41)	7 (23)	
Extramural vascular invasion	*No*		90 (59)	22 (71)	0.22
	*Yes*		62 (41)	9 (29)	
Perineural invasion	*No*		113 (74)	27 (87)	0.13
	*Yes*		39 (26)	4 (13)	
*Tumour interface markers*					
Peritumoural budding	*Grade I*		122 (80)	28 (90)	0.32
	*Grade II*		26 (17)	2 (6)	
	*Grade III*		4 (3)	1 (3)	
Peritumoural budding	*No (Grade I)*		122 (80)	28 (90)	0.18
	*Yes (Grade II/III)*		30 (20)	3 (10)	
Primary CRC growth pattern	*Expanding*		72 (47)	18 (58)	0.28
	*Infiltrative*		80 (53)	13 (42)	
Stroma type	*Immature*	*16 (9)*^a^	18 (13)	2 (7)	0.02
	*Intermediate*		33 (24)	1 (3)	
	*Mature*		87 (63)	26 (90)	
Non-mature stroma	*No*		101 (66)	28 (90)	0.008
	*Yes*		51 (34)	3 (10)	
*Immunological markers*					
TIL density in %—*median [IQR]*			10.0 [5.0, 15.0]	15.0 [10.0, 20.0]	0.02
Median-to-low TIL density	*No*		47 (31)	18 (58)	0.004
	*Yes*		105 (69)	13 (42)	
Crohn's-like lymphoid reaction	*No*		21 (14)	0 (0)	0.03
	*Yes*		131 (86)	31 (100)	

^a^Only assessable in case of (near) extramural invasion (i.e., pT3-4)

*CRC* colorectal cancer, *IQR* interquartile range, *TIL* tumour-infiltrating lymphocyes

### Classical markers

Poorly differentiated (G3) tumours (5% vs 0%), right-sided tumours (30% vs 16%), pT4-stage (16% vs 6%), positive lymph nodes (62% vs 48%), and tumour deposits (21% vs 10%) were more common in patients with corresponding non-desmoplastic versus desmoplastic CRLM, but none of these differences reached statistical significance (*p*-values of 0.22, 0.11, 0.15, and 0.16, respectively, Table 2).

### Invasion markers

Invasion, either lymphovascular (41% vs 23%), extramural venous (41% vs 29%), or perineural (26% vs 13%), was more prevalent in patients with non-desmoplastic versus desmoplastic CRLM, but none of these differences reached formal statistical significance (*p*-values of 0.06, 0.22, and 0.13, respectively, Table 2).

### Tumour interface markers

Peritumoural budding (grade II/III vs I: 20% vs 10%) and an infiltrative CRC growth pattern (53% vs 42%) were also more common for patients with corresponding non-desmoplastic versus desmoplastic metastases, but again these differences did not reach statistical significance (*p* = 0.18 and *p* = 0.28 respectively, Table 2). Of the patients with non-desmoplastic CRLM, 18 (13%) had an immature, 33 (24%) an intermediate, and 87 (63%) a mature fibrotic stroma type, whereas this was 2 (7%), 1 (3%), and 26 (90%) respectively for patients with desmoplastic CRLM, a difference that was statistically significant (*p* = 0.02, Table 2). Consequently, a non-mature (i.e., immature or intermediate) stroma was significantly more often observed in non-desmoplastic compared to desmoplastic patients (34% vs 10%, *p* = 0.008, Table 2).

### Immunological markers

Crohn’s-like lymphoid reaction was observed in 131 (86%) of the patients with non-desmoplastic CRLM versus in all 31 (100%) of the patients with desmoplastic CRLM (*p* = 0.03, Table 2). The TIL density (median [IQR]) was significantly lower for the non-desmoplastic (10% [5%-15%]) versus desmoplastic (15% [10%-20%]) patients (*p* = 0.02, Fig. 3B). Consequently, a median-to-low (≤ 10%) TIL density was significantly more common in the non-desmoplastic (*n* = 105 [69%]) compared to desmoplastic (*n* = 13 [42%]) group (*p* = 0.004, Table 2).

### Cumulative prevalence and HGP prediction

Overall, unfavourable CRC histopathology was significantly more prevalent in non-desmoplastic compared to desmoplastic patients with a median (IQR) of 4 (3–6) versus 2 (1–3.5) unfavourable features observed, respectively (*p* < 0.001, Fig. 3C). The results of the uni- and multivariable binary logistic regression analyses to predict the HGP are reported in Table 3. Differentiation grade and Crohn’s-like lymphoid reaction could not be analysed using logistic regression given absent cases in the desmoplastic group. Upon univariable analysis the CRLM characteristics disease-free interval and diameter, and all primary CRC histopathology features except extramural vascular invasion and CRC growth pattern had a *p*-value below 0.2 and were considered for multivariable analysis (Table 3). Of all 11 predictors in the multivariable model, only the diameter of the largest CRLM and TIL density proved independent predictors for a desmoplastic HGP, with an OR (95%CI) of 0.55 (0.37–0.82) for each additional cm and 1.60 (1.01–2.54) per 10% increase in TIL density, respectively (Table 3). The multivariable logistic regression model achieved an AUC of 0.83 to predict the HGP.

**Table 3:** Uni- and multivariable logistic regression analysis on the desmoplastic growth pattern

	Univariable		Multivariable (*n* = 182)	
	OR [95%CI]	*p*-value	OR [95%CI]	*p*-value
*CRLM characteristics*				
Disease-free interval^a^ (cont.)—*months*	0.98 [0.96–1.01]	0.18	0.99 [0.96–1.02]	0.54
Number of CRLM (cont.)	0.93 [0.72–1.21]	0.60	-	-
Diameter of largest CRLM (cont.)—*cm*	0.58 [0.41–0.81]	< 0.01	0.55 [0.37–0.82]	< 0.01
Preoperative CEA (cont.)—*100 µg/L*	0.42 [0.10–1.68]	0.22	-	-
Extrahepatic disease—*yes vs no*	0.28 [0.04–2.22]	0.23	-	-
*Classical markers*				
Right-sided tumour—*yes vs no*	0.44 [0.16–1.23]	0.12	0.50 [0.16–1.60]	0.24
pT4-stage—*yes vs no*	0.35 [0.08–1.56]	0.17	0.43 [0.08–2.26]	0.32
Positive lymph nodes—*yes vs no*	0.58 [0.27–1.26]	0.17	0.65 [0.25–1.69]	0.38
Tumour deposits—*yes vs no*	0.40 [0.11–1.41]	0.15	0.48 [0.08–2.95]	0.43
*Invasion markers*				
(lympho-)vascular invasion—*yes vs no*	0.42 [0.17–1.04]	0.06	0.68 [0.22–2.12]	0.51
Extramural vascular invasion—*yes vs no*	0.59 [0.26–1.38]	0.22	-	-
Perineural invasion—*yes vs no*	0.43 [0.14–1.30]	0.14	1.11 [0.28–4.46]	0.89
*Tumour interface markers*				
Peritumoural budding—*Grade II/III vs I*	0.44 [0.12–1.53]	0.19	0.51 [0.13–2.02]	0.34
CRC growth pattern—*Infiltrative vs expanding*	0.65 [0.30–1.42]	0.28	-	-
Non-mature stroma—*yes vs no*	0.21 [0.06–0.73]	0.01	0.29 [0.07–1.25]	0.10
*Immunological markers*				
TIL density (cont.)—*10%*	1.69 [1.15–2.47]	< 0.01	1.60 [1.01–2.54]	0.05

Abbreviations in alphabetical order: *Cont.* entered as continous variable, *CEA* carcinoembryonic antigen, *CI* confidence interval, *CRC* colorectal cancer, *CRLM* colorectal liver metastasis, *OR* odds ratio, *TIL* tumour-infiltrating lymfocyte.

^a^Between resection of primary tumor and detection of CRLM

#### Survival

**Fig. 4:**
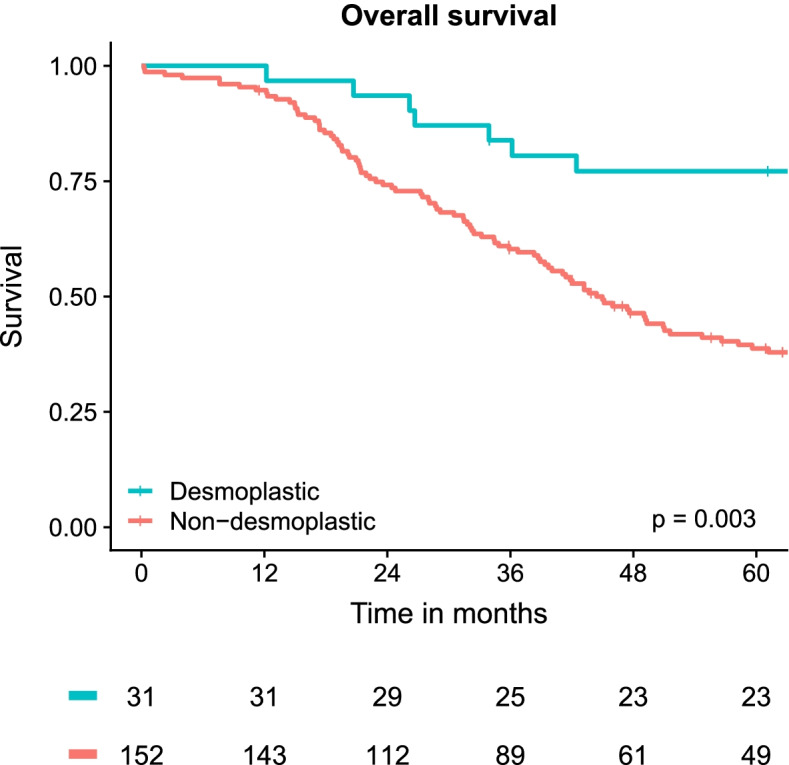
Kaplan–Meier analysis on overall survival following resection of colorectal liver metastasis stratified by histopathological growth pattern. The *p*-value represents the results of the overall log-rank test

Patients with a desmoplastic HGP had a significantly longer OS following resection of CRLM with an estimated 5-year (95%CI) survival of 77% (64–94%) compared to 39% (31–48%) for non-desmoplastic (*p* = 0.003, Fig. 4). Univariable OS regression analysis revealed four CRLM characteristics (age at resection, number of CRLM, extrahepatic disease, and the HGP) and nine CRC histopathology features (right-sided, differentiation grade, pT4-stage, positive lymph nodes, tumour deposits, [lympho]-vascular, extramural vascular and perineural invasion, and the CRC growth pattern) with a *p*-value below 0.2 and were considered for multivariable analysis (supplementary file 2). Of these, only age at resection, the HGP, and a right-sided tumour proved independent predictors for survival, with adjusted HRs (95%CI) of 1.33 [1.10–1.62] per 10-year age increase, 1.97 [1.10–3.53] for a non-desmoplastic HGP, and 1.86 [1.23–2.83] for right-sided tumours, respectively (supplementary file 2).

## Discussion

The present study evaluated the relationship between thirteen established CRC histopathology markers and the HGPs of corresponding CRLM in a cohort of 183 resected patients. For all markers, unfavourable CRC histopathology was more frequent for patients with corresponding non-desmoplastic CRLM. While many of these individual marker differences did not reach statistical significance, the cumulative prevalence of unfavourable CRC histopathology was significantly higher in the non-desmoplastic patients.

At least two other studies have previously evaluated CRC histopathology in relation to the CRLM growth pattern phenotype. The more recent study by Wu et al. evaluated 29 patients and compared primary CRC histopathology between 15 patients with a predominant (i.e. > 50%) desmoplastic versus 14 with a predominant replacement pattern [24]. The study significantly associated the predominant replacement group (i.e. non-desmoplastic) with higher peritumoural budding grades, an infiltrative CRC growth pattern, and *absent* Crohn’s disease-like response. Rajaganeshan and colleagues evaluated in 55 patients the relationships between primary CRC growth pattern and CRLM encapsulation, the latter defined as > 50% fibrous capsule formation separating tumour from stroma (i.e. > 50% desmoplastic), and also significantly associated an infiltrative CRC growth pattern with corresponding non-encapsulated (i.e. non-desmoplastic) CRLM [25]. An important distinction with these studies lies in the classification of the HGP, as both applied a 50% predominance cut-off as opposed to the newly recommended Rotterdam criteria of entirely desmoplastic versus otherwise. There is compelling evidence from both a prognostic [8, 9] and immunologic [26] standpoint that it is this distinction between desmoplastic and non-desmoplastic that delineates clinical relevance. Results of studies applying predominance cut-offs are therefore difficult to extrapolate in light of this new classification, as the predominant desmoplastic groups (i.e. > 50%) are by definition, and based on previous studies on HGP distribution [8, 9], actually for more than half composed of non-desmoplastic cases. Nevertheless, taking all current evidence as a whole, non-desmoplastic CRLM have repeatedly been associated with unfavourable CRC histopathology, something also evident from the relationship with lymph node positivity observed in multiple large cohort studies evaluating the HGPs [8, 9]. The current study confirms these associations and adds further compelling evidence of the relationship between CRC histopathology and the HGPs of corresponding CRLM.

While not all individual marker differences demonstrated a statistically significant association, both the immunology markers TIL-density and Crohn’s-like lymphoid reaction did, with higher TIL-densities and increased Crohn’s-like lymphoid reaction observed in the patients with desmoplastic CRLM. Both markers have been associated with a survival benefit after resection of primary CRC and are thought to reflect anti-tumour (host) immunity, with increased TIL-densities and Crohn’s-like reaction indicative of a more effective antitumour host-response [27]. The cellular composition and structure of these Crohn’s-like lymphoid aggregates is similar to secondary lymphoid organs, and studies have linked these structures with increased TIL infiltration [27] and cytotoxic gene expression signatures [28], indicating that these lymphoid aggregates are functional components of the adaptive anticancer immune response in CRC [29]. These results therefore suggest an increased adaptive immune response in the originating primary colorectal cancers of patients who develop corresponding desmoplastic liver metastasis. Evaluations of the immune microenvironment of CRLM have revealed similar results, that is an increased and distinctly cytotoxic immune response observed in the desmoplastic HGP [26, 30]. This now associates the desmoplastic phenotype with increased antitumor immunity in both the originating primary colorectal tumour, as well as the localised liver metastasis microenvironment, hinting at a degree of systemic anti-tumour response in these patients. Taken together with recent associations between microsatellite instability-high colorectal cancers, an actionable target for immunotherapy in stage I-III [31] and IV [32] CRC, and desmoplastic CRLM [9], there is growing evidence to suggest that (systemic) anticancer immunity plays an important role in the underlying biology of the HGPs. While this infers causality, the fact that patient-derived xenografts in SCID-beige mouse with defective T- B- and NK-cell activity have been successful in producing liver lesions with an identical HGP as the donor patient metastasis following intrahepatic transplantation however argues against the HGPs as a solely immunologically driven process [33]. In addition, liver metastases appear to suppress systemic immunity in general, with immunotherapies appearing less effective in the presence of hepatic dissemination [34]. Whether the HGP of a liver metastasis influences the degree of systemic immunosuppression remains to be explored.

Of the other markers evaluated the extramural fibrotic stroma type was also significantly associated with the corresponding liver metastasis HGP. The prognostic impact of the extramural stroma type after resection of primary CRC has been demonstrated in multiple retrospective series, and more recently within a prospective phase III trial [20, 35, 36]. In addition, characterisation of the extramural stroma type of primary CRC also proved prognostic for survival following resection of corresponding CRLM [37]. Although this could not be validated in the current study, which found a univariable HR (95%CI) of 1.06 (0.72–1.57) for a non-mature stroma type (supplementary file 2). Of the three types recognized, the immature type has the worst prognosis, followed by the intermediate type, and with the most favourable prognosis observed in the mature type. There are several arguments to indicate that the non-mature stroma types (i.e., immature and intermediate) reflect a state of activated epithelial-mesenchymal transition (EMT) promoting invasive and migratory cancer properties. Both have for instance been associated with higher degrees of tumour budding [38], a known phenotype of EMT-related gene expression [39], which was also true in this study cohort (data not shown). The non-mature stroma’s, and notably the characterising myxoid stroma of the immature type, also exhibit increased extracellular matrix component depositions amongst which fibronectin [35], a known activator of EMT [40]. In addition, the defining eosinophilic collagen bundles of the intermediate type are similarly observed in keloids, a microenvironment characterised by overexpression of fibroblast associated growth factors including transforming growth factor β (TGF-β) [41]. Taking the position that these are alike, TGF-β, a well-recognised EMT stimulating factor, is likely to be upregulated in the intermediate type [42]. The association between non-desmoplastic CRLM and these non-mature stroma types suggests increased EMT activation in the primary tumours of these patients. Indeed, other histomorphological signs of invasive and migratory growth potential such as vessel invasion and peritumoural budding were also exclusively more frequent in the primary tumours of corresponding non-desmoplastic metastases, albeit not statistically significantly so.

Besides the association with individual histopathology markers, this study found that overall, unfavourable CRC histopathology was associated with non-desmoplastic CRLM given the significantly higher cumulative prevalence of unfavourable charactheristics observed in these patients. This suggests that the information contained in primary CRC histology may be exploited to predict the HGP. And indeed, a multivariable model containing 9 primary CRC characteristics and 2 CRLM characteristics achieved good performance (AUC = 0.83) to predict the HGP. Of all CRC markers included only TIL density however proved an independent predictor, with all other characteristics failing to reach statistical significance. When interpreting these results it is important to consider that almost all markers had an estimated odds-ratio around the 0.5 mark, but were insignificant as a result of a large uncertainty of this estimate, i.e. wide confidence intervals. The model therefore predominantly demonstrates that prediction of the HGP using both CRLM and CRC histopathology characteristics could be feasible, but that the current sample-size is insufficient to properly assess the individual predictive properties of all included markers. Something also highlighted by the fact that not all markers could be included in these regression analyses given absent cases in the desmoplastic group. As such, the results of this study should serve more as a stepping stone for a deep-learning digital-pathology approach in a larger cohort. Several deep-learning models already exist for the automated detection and classification of individual markers, for instance peritumoural budding [43], TIL density [44], and the fibrotic stroma type [45]. Additionally, studies have shown deep-learning on histopathology capable of predicting relevant outcomes in a hypothesis-free manner, i.e., not training the model to predict specific markers but instead let the model identify relevant features itself for accurate prediction of the outcome of interest. Examples are the prediction of survival after resection of both primary CRC [46] and CRLM [47], and such a hypothesis-free approach could also be considered to predict the HGP of CRLM on digitalised slides of the corresponding primary CRC tumour. Such a study would require a large number of digitalised slides of resected CRC and corresponding CRLM from multiple independent cohorts. Collection of such datasets may therefore be worthwhile to pursue.

The survival analysis in light of the HGP, other CRLM and patient characteristics, and all included CRC histopathology markers demonstrated the HGP as one of three independent predictors for overall survival upon multivariable analysis. But again, the lack of statistical power was evident, as multiple markers – Including established prognosticators such as lymph-node positivity – demonstrated clinically relevant estimates but failed to reach statistical significance based on the estimate uncertainty (supplementary file 2). Nevertheless, these results support the clinical relevance of this biomarker, similarly to the two previous retrospective series on which this cohort is partly based [8, 9]. In addition, a recent study of over 4000 patients evaluating survival after CRLM surgery in light of new biomarkers including the HGP found it to be amongst the independent prognosticators with the largest impact on survival, only being equalled by KRAS and BRAF mutational status, respectively [6].

The results of this study have to be considered in light of its limitations. Most importantly the inadequate sample-size to detect small to moderate individual marker differences, increasing the likelihood of type 2 statistical errors and not allowing for sufficiently powered multivariable regression analysis increasing the risk for overfitted models. For example, as a general rule of thumb it is advised that the number of variables in a multivariable regression model should not exceed 10% of the total number of events. In our study this relates to the 31 patients with a desmoplastic HGP, and therefore the multivariable model should preferably be limited to less than 4 predictors instead of the 11 actually included. The results should therefore be interpreted with caution and serve more as a proof of concept for a future validation or follow-up study. In addition, this study only included patients who did not receive any chemo- or radiotherapy prior to both CRC and CRLM surgery. While this is from an analysis standpoint not a limitation per se, the applicability of the results is lessened as most patients who undergo surgical resection of CRLM are generally treated with preoperative systemic chemotherapy which can alter the HGP, with higher rates of the desmoplastic HGP found after chemotherapy [12]. Interestingly, within the 119 patients with metachronous CRLM, those who received adjuvant systemic chemotherapy following CRC resection more often had a non-desmoplastic HGP. This is likely the result of confounding by indication, as both node-positivity and pT4 stage were more common in the patients with a non-desmoplastic HGP. Moreover, all patients in the current study did not receive any chemotherapy in the six months prior to CRLM diagnosis. Another limitation is the lack of data on molecular characteristics such as KRAS and BRAF mutational status. Unfortunately, many of the patients in this study were operated on before the implementation of these genetic markers into routine clinical practice, and data on these markers was consequently only available for less than one-fifth. Previous studies however did not find an association between these markers and the HGP of CRLM [9], and found the prognostic impact independent of these genetic alterations [6, 9], but in-depth genetic association studies remain lacking. Lastly, all histopathological assessment was observer-based, while an increasing number of automated assessments are available for more reproducible and precise estimations. These limitations underscore the need for external validation not limited to treatment-naive patients, and ideally with observer-independent methods of assessment.

In conclusion, our results associate primary colorectal cancer histopathology with the histopathological growth patterns of corresponding colorectal liver metastases, and may aid in their preoperative determination. In addition, it associates the desmoplastic phenotype with an increased host-immune response, and the non-desmoplastic type with histomorphological evidence of epithelial-mesenchymal transition at the primary tumour microenvironment level.

## Supplementary Information

**Additional file 1.** Handbook histopathological features CRC – HGP.

**Additional file 2. Supplementary File 2.** Uni- and multivariable Cox regression analysis for overall survival.

## Data Availability

All data and digital slides are available upon request and at the discretion of the corresponding author.
