# Supplementary material for: The relationship between primary colorectal cancer histology and the histopathological growth patterns of corresponding liver metastases
Source: BMC Cancer. 2022 Aug 22;22:911. doi: 10.1186/s12885-022-09994-3 (PMC9394040; doi:10.1186/s12885-022-09994-3)
Supplement: Supplementary file 1 — Additional file 1. Handbook histopathological features CRC – HGP. [file 12885_2022_9994_MOESM1_ESM.pdf]

Handbook histopathological features CRC – HGP

Contents

Tumour deposits (TD) 2

Lymphovascular invasion (LVI) 3

Extramural vascular/venous invasion (EMVI) 4

Perineural invasion (PNI) 5

Peritumoural budding (PTB) 6

Expanding growth pattern (EGP) 9

Infiltrating growth pattern (IGP) 10

Stroma type 11

- Immature stroma type (type A) 12
- Intermediate stroma type (type B) 13
- Mature stroma type (type C) 14

Crohn's-like lymphoid reaction (CLR) 15

Tumour infiltrating lymphocytes (TIL) 18

References 21

Tumour deposits (TD)

Definition:
Tumour deposits are defined as discrete tumour nodules of any shape, contour or size that lack associated lymph node tissue, vascular structures or neural structures found within the lymph drainage area of the primary carcinoma. (1)

Examination:
AJCC 8 clarifies the interpretation of discrete tumour nodules found within the lymph drainage area of a primary colon or rectal carcinoma. Nodules containing no identifiable lymph node tissue or vascular/neural structures should be considered tumour deposits. The shape, contour, and size of the deposit are not considered in these designations. (1) The presence of tumour deposits is evaluated and the number of tumour deposits is counted.

Photo:

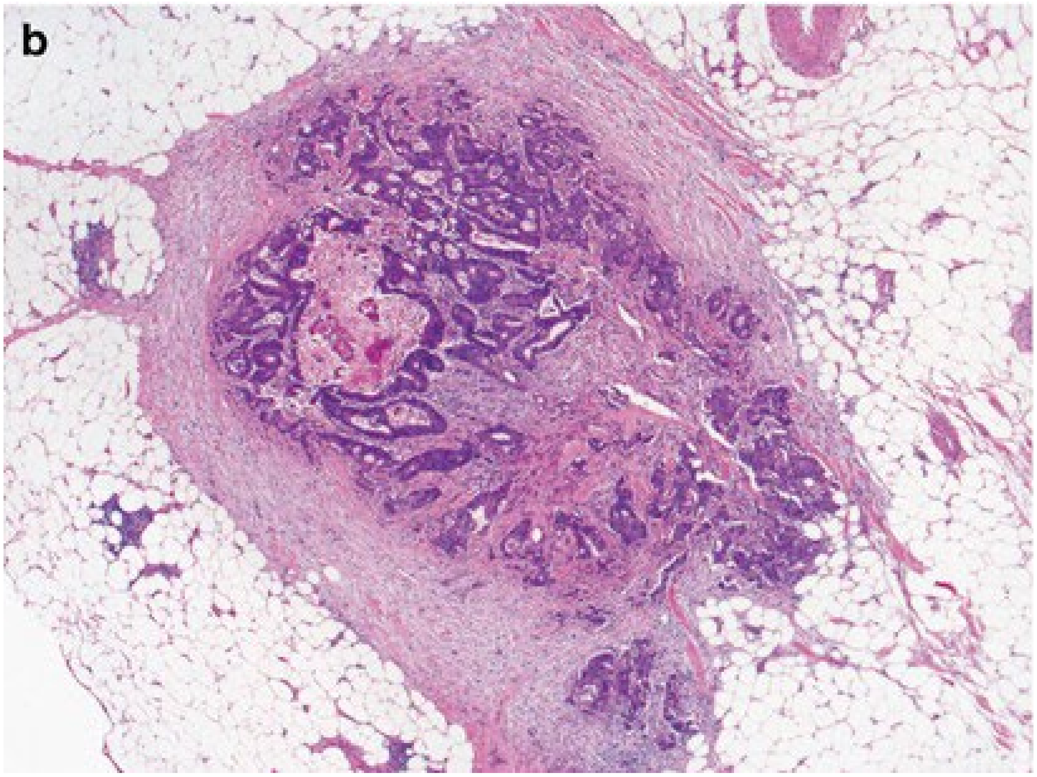

Tumor deposit. (2)

Lymphovascular invasion (LVI)

Definition:
Lymphovascular invasion (LVI) is defined as the presence of tumour cells within a definite endothelial-lined space (lymphatics or blood vessels) in the colon/rectum surrounding invasive carcinoma. (3)

Examination:
At least 3 blocks of tumour (optimally 5 or more blocks) should be submitted. A single H&E stained section from each block should be examined for venous or lymphatic vessel invasion. (3)

Photo:

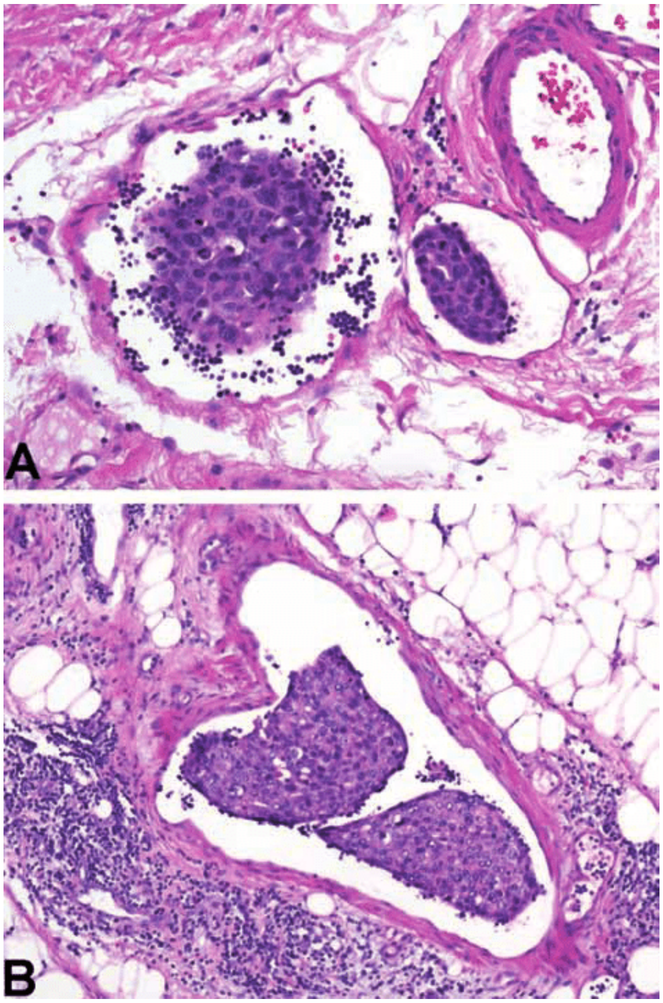

A-B: Positive lymphovascular invasion (LVI). (4)

Extramural vascular/venous invasion (EMVI)

Definition:
Extramural vascular/venous invasion (EMVI) is defined as the presence of large vessel invasion beyond the muscularis propria. (5)

Examination:
Vessels with a clear endothelial lining and thin walled should be considered lymphatic (small), whereas large vessels (venous) includes all with a muscular wall. In suspicious cases, sections at multiple levels or sections with elastin staining have to be used to confirm venous invasion. Venous invasion beyond the muscularis propria is considered extramural vascular invasion. (5)

Photo:

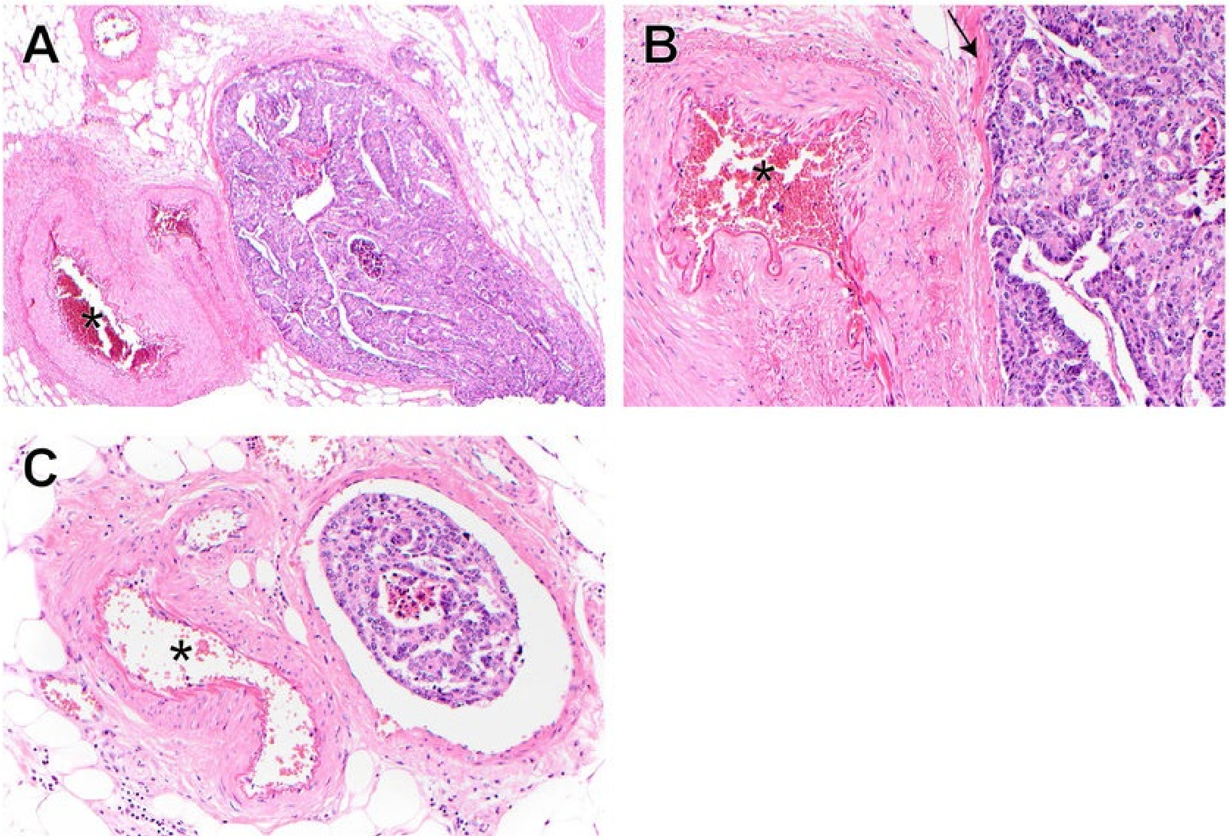

A: EMVI in a large vein. B: Higher magnification of A, with highlight to vein wall (arrow). C: EMVI in a smaller vein. (5)

Perineural invasion (PNI)

Definition:
Perineural invasion (PNI) is assessed positive when cancer cells are observed inside the nerve sheath, or when at least 33% of the nerve periphery is surrounded by cancer cells. (6)

Examination:
See definition above. When perineural invasion in found in any slide, it is considered positive.

Photo:

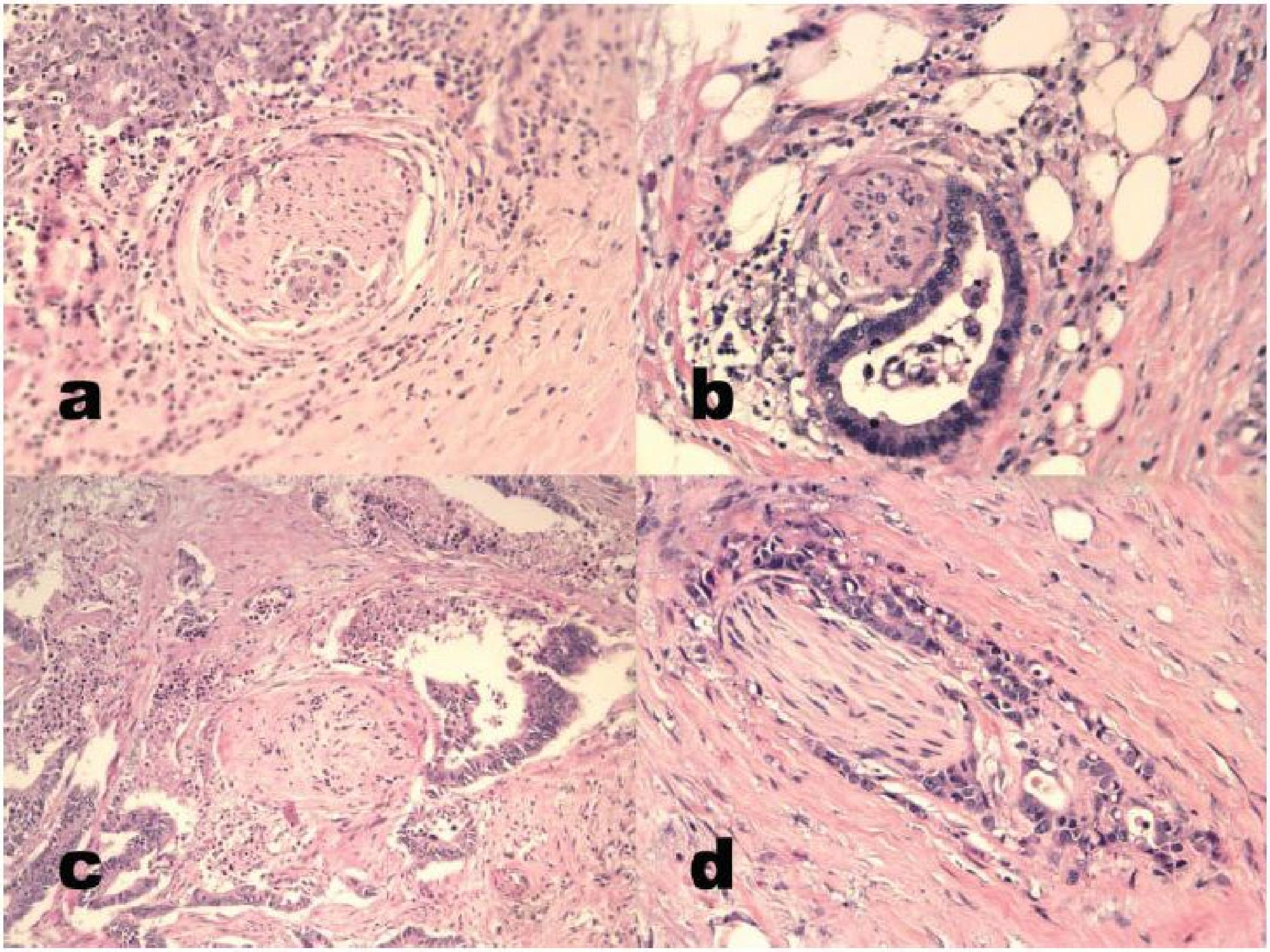

A: Tumor cells located inside the nerve sheath. B: Glandular element inside the nerve sheath. C and D: Nerves with at least 33% of the nerve periphery surrounded by cancer cells. (6)

Peritumoural budding (PTB)

Definition:
Peritumoural budding (PTB) is defined as a single tumour cell or a cell cluster of up to 4 tumour cells at the tumour front. (7)

Examination:
See flowchart (figure 4) below.
The prodecure is followed proposed by the ITBCC 2016. (7)
Define the field (specimen) area for the 20x objective lens of the microscope based on the eyepiece field number (FN) diameter. Select the H&E slide with greatest degree of budding at the invasive front.
Scan 10 individual fields at medium power (10x objective) to identify the 'hotspot' at the invasive front.
Count tumour buds in the selected 'hotspot' (20x objective). Divide the bud count by the normalization factor to determine the tumour bud count per 0,785 mm^2.

Normalization factor depending on eyepiece field number (FN) diameter:

Objective magnification: 20		
Eyepiece FN diameter (mm)	Specimen area (mm2)	Normalization factor
18	0.636	0.810
19	0.709	0.903
20	0.785	1.000
21	0.866	1.103
22	0.950	1.210
23	1.039	1.323
24	1.131	1.440
25	1.227	1.563
26	1.327	1.690

Photos:

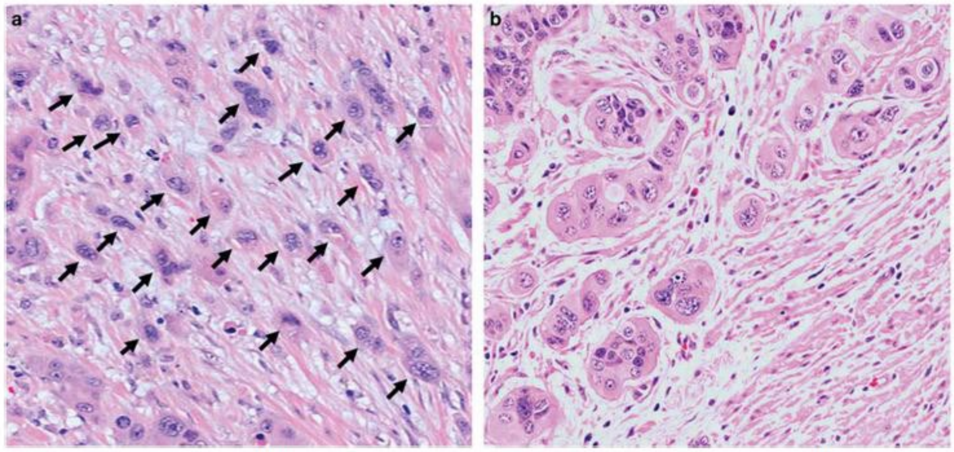

A: Example of tumour budding that is defined as a single tumour cell or tumour cell clusters at up to four cells. B: example of poorly differentiated clusters that are defined as five tumour cells or more. (7)

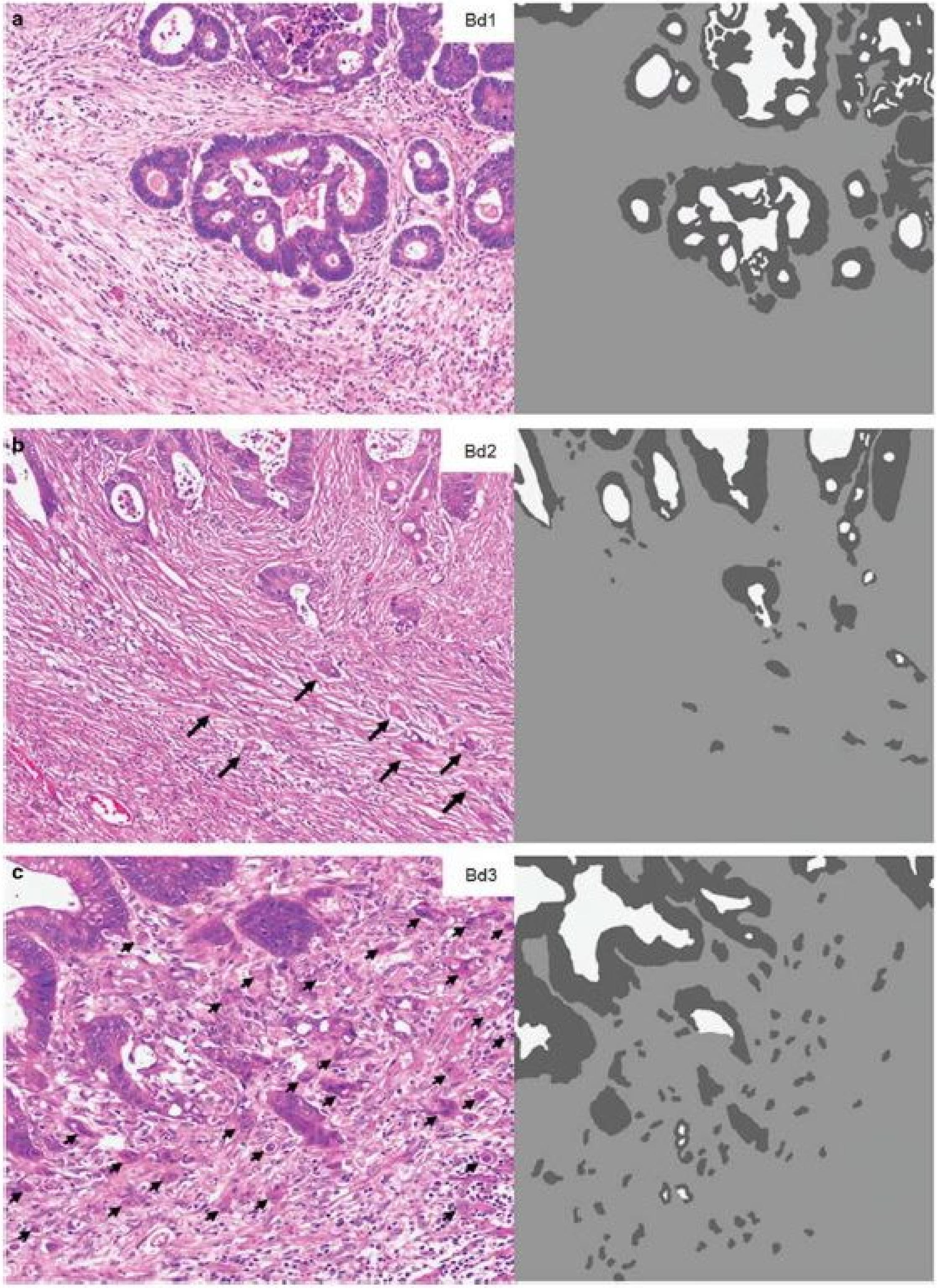

Examples of different tumour budding grades at the invasive front of colorectal cancer based on the ITBCC 2016. A: Low tumour budding grade. B: Intermediate tumour budding grade. C: High tumour budding grade. (7)

1 **Define the field (specimen) area for the 20x objective lens of your microscope based on the eyepiece field number (FN) diameter**

Objective magnification: 20		
Eyepiece FN Diameter (mm)	Specimen Area (mm2)	Normalization Factor
18	0.636	0.810
19	0.709	0.903
20	0.785	1.000
21	0.866	1.103
22	0.950	1.210
23	1.039	1.323
24	1.131	1.440
25	1.227	1.563
26	1.327	1.690

---

2 **Select the H&E slide with greatest degree of budding at the invasive front**

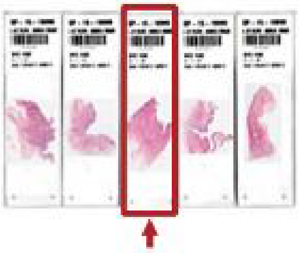

---

3 **Scan 10 individual fields at medium power (10x objective) to identify the "hotspot" at the invasive front**

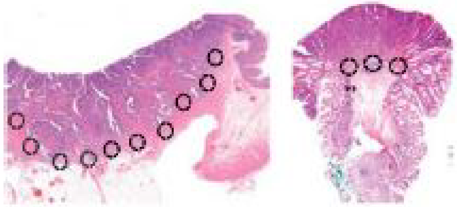

For surgical resection specimens, scan 10 fields

For pT1 endoscopic resections (usually <10 fields available), scan all

---

4 **Count tumor buds in the selected "hotspot" (20x objective)**

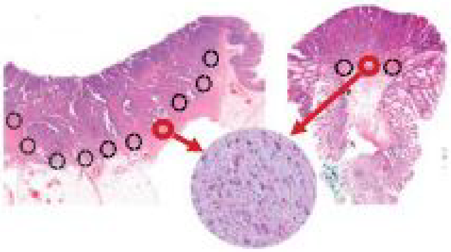

Selected hotspot indicated in red

---

5 **Divide the bud count by the normalization factor (figure 2) to determine the tumor bud count per 0.785mm^2**

Select the budding [Bd] category based on bud count and indicate the absolute count per 0.785mm^2 (see reporting example)

Tumor bud count per 0.785 mm^2 = Bud count (20x objective) / Normalization factor*

**Bd1** (low):	0-4 buds	per 0.785 mm^2
**Bd2** (intermediate):	5-9 buds	
**Bd3** (high):	≥10 buds	

Reporting example:
Tumor budding: Bd3 (high), count 14 (per 0.785 mm^2)

Procedure proposed by the ITBCC 2016 for reporting tumour budding in colorectal cancer in daily diagnostic practice. (7)

Expanding growth pattern (EGP)

Definition:
Colorectal carcinomas are defined as 'expanding' when the invasive margin is pushing or reasonably well circumscribed. (8)

Examination:
The invasive front of the primary cancer is classified as ‘expanding growth pattern’ (EGP) based on the predominant morphology, as defined by Jass et al, where the ‘expanding’ type had been described as the pushing growth type of adenocarcinoma. (8) The amount of EGP present at the invasive front is noted in percentage of the total invasive front.

Photo:

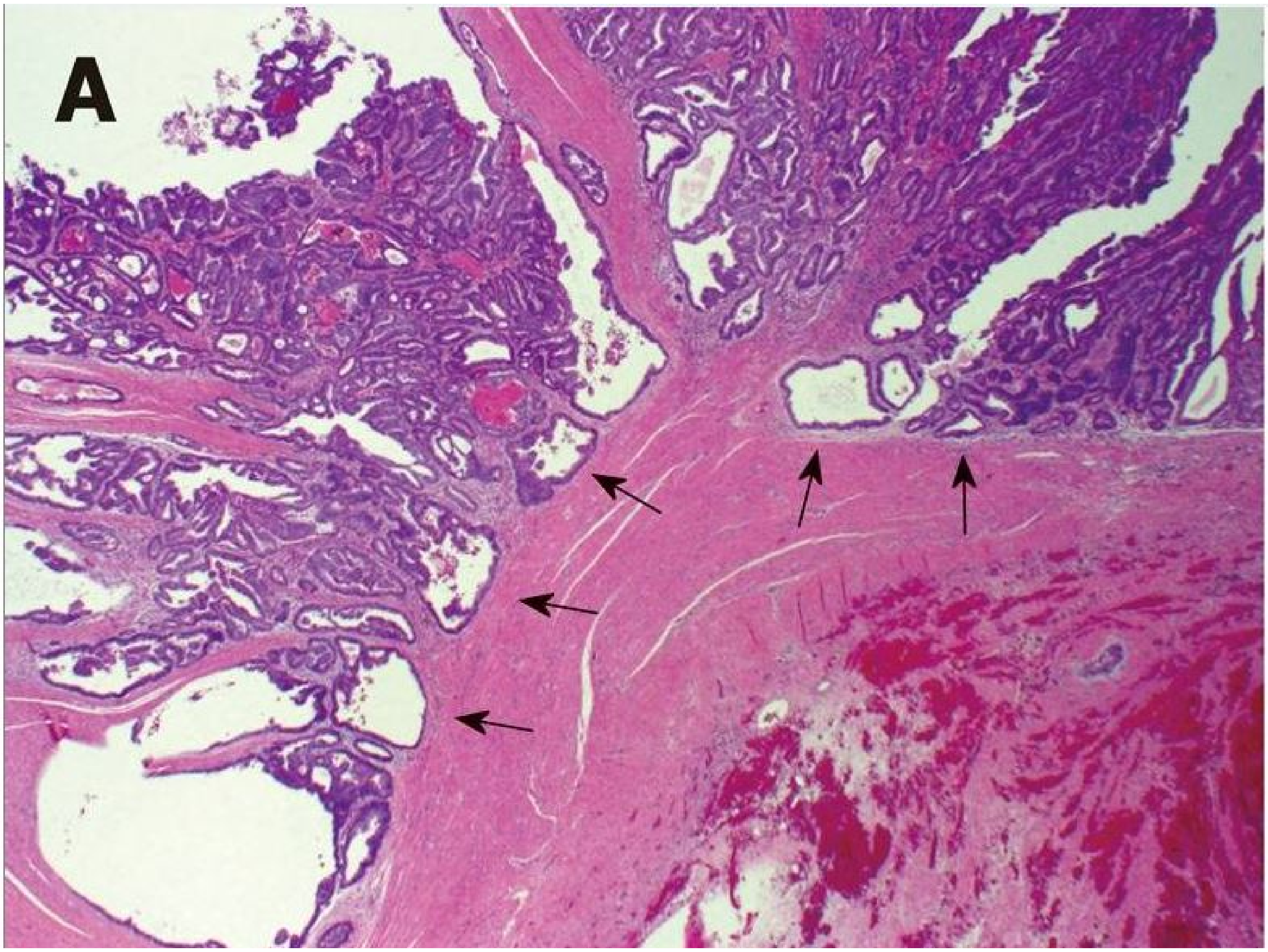

Colorectal carcinoma with an expanding growth pattern where the tumour pushes the surrounding stroma with a sharp dividing interface. (9)

Infiltrating growth pattern (IGP)

Definition:
Colorectal carcinomas are defined as 'infiltrating' when the tumour invaded in a diffuse manner with widespread penetration of normal tissues.

Examination:
The invasive front of the primary cancers were classified as infiltrating growth pattern (IGP) based on the predominant morphology, as defined by Jass et al, where the infiltrating type had been described as the wide spread streaming form of adenocarcinoma. (8) The amount of IGP present at the invasive front is noted in percentage of the total invasive front.

Photo:

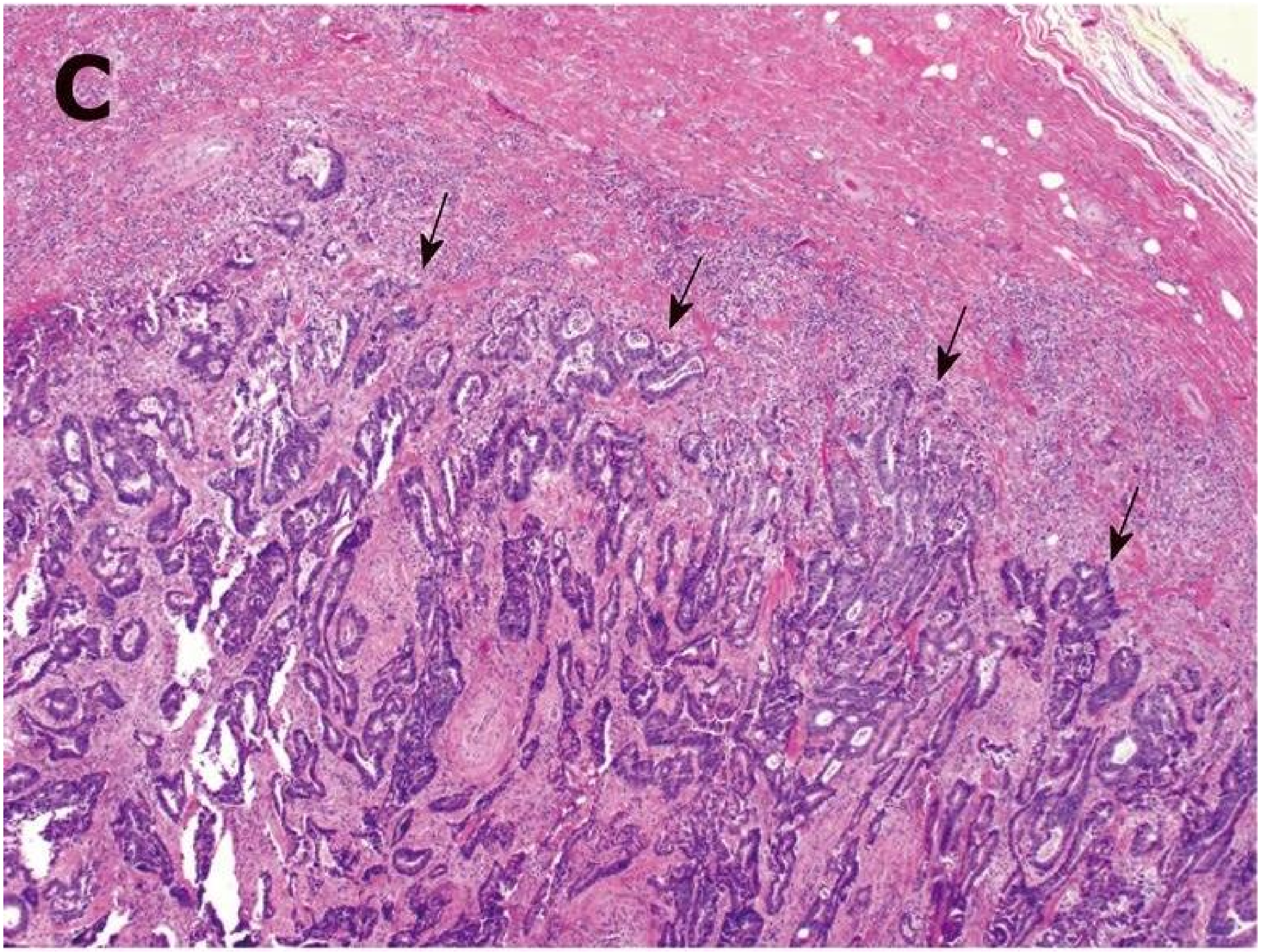

Colorectal carcinoma with an infiltrating growth pattern where tumour glands penetrate the surrounding stroma with an irregular interface. (9)

Stroma type

See flowchart below for interpretation.

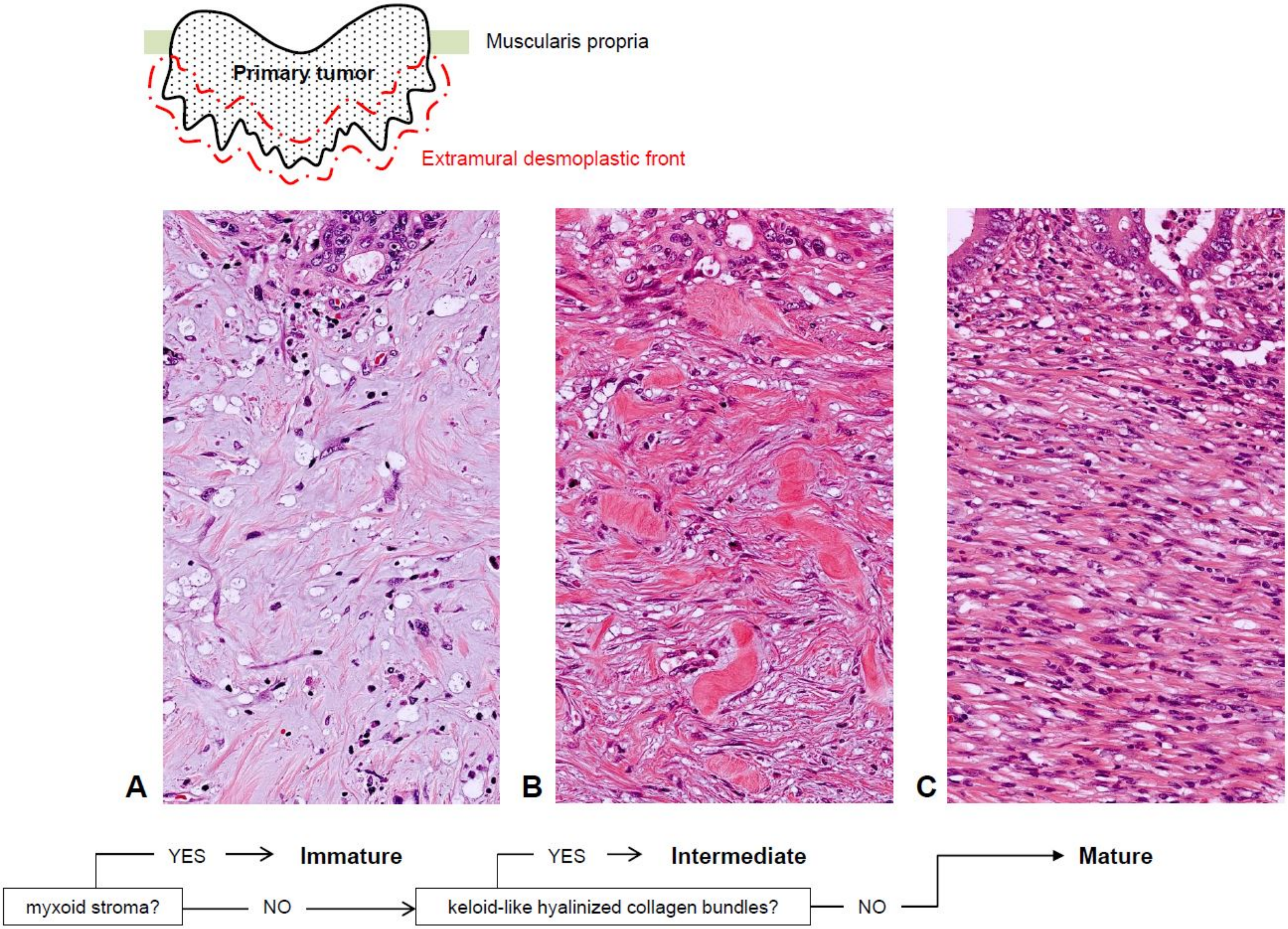

Examples of the different types of stroma identified at the extramural front. (10)

Immature stroma type (type A)

Definition:
Tumours with a myxoid stroma are defined as ' immature'. (11)

Examination:
The type of stroma is classified based on the predominant morphology of the stroma, where ‘immature’ (myxoid) stroma is defined as stroma with an amorphous mucinous substance, typically composed of a slightly basophilic or amphophilic, vacuolated extracellular material among the collagen fibers present in at least one field as viewed through a 40x objective lens. (11)

Photo:

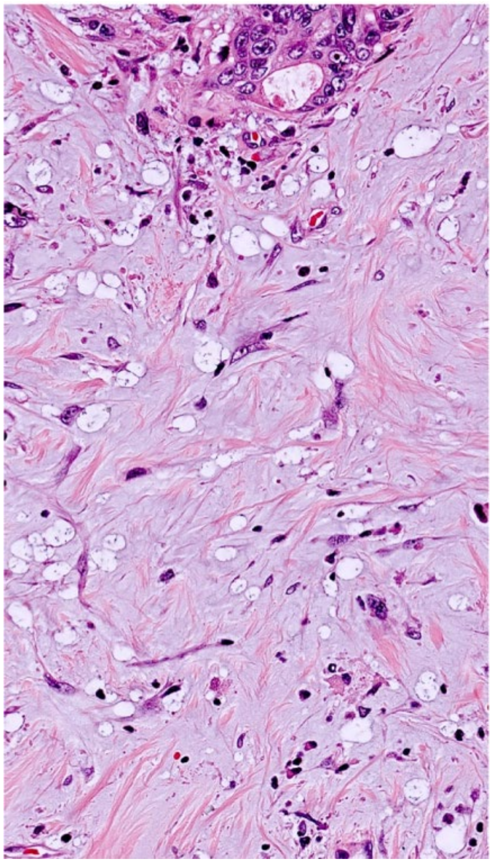

Myxoid stroma, an amorphous stromal substance comprising basophilic extracellular matrix. (10)

Intermediate stroma type (type B)

Definition:
Tumours without a myxoid stroma, but with a keloid-like collagen in the fibrotic stroma was defined as 'intermediate'. (11)

Examination:
The type of stroma is classified based on the predominant morphology of the stroma, where ‘intermediate’ (keloid-like collagen) stroma is defined as hyalinized thick bundles of hypocellular collagen with bright eosinophilic hyalinization. (11)

Photo:

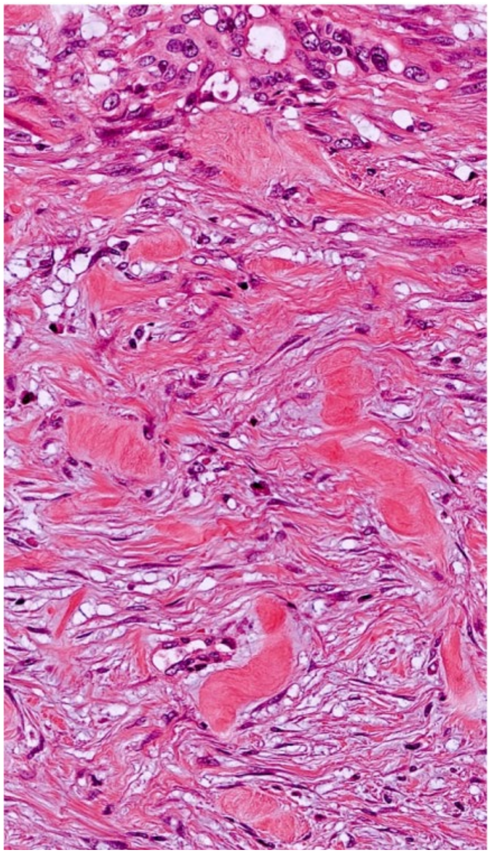

Intermediate stroma, characterized by thick keloid-like hyalinised collagen bundles. (10)

Mature stroma type (type C)

Definition:
Tumours that were only composed of fine mature collagen fibers stratified into multiple layers in all reactive fibrous zones were defined as 'mature'. (11)

Examination:
The type of stroma is classified based on the predominant morphology of the stroma, where 'mature' stroma is defined as fine mature collagen fibers stratified into multiple layers in all reactive fibrous zones. (11)

Photo:

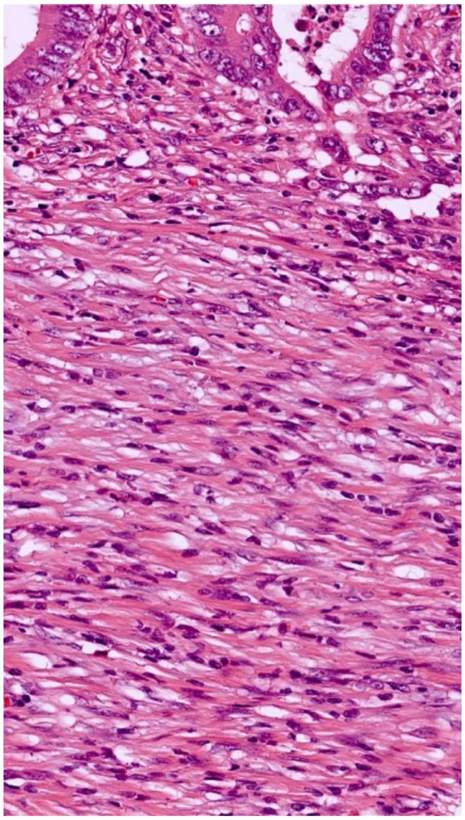

Mature stroma type, characterized by only fine mature collagen fibres stratified into multiple layers. This stroma type does not contain myxoid stroma or keloid-like collagen. (10)

Crohn's-like lymphoid reaction (CLR)

Definition:
Crohn's-Like Lymphoid Reaction (CLR) is defined as the presence of peritumoural lymphoid aggregates (LAs). (12) For evaluation we use the presence of the LAs and determine the number of LAs.

Examination:
We used the evaluation method of Ueno et al (12).
Nodular lymphoid aggregates lining the tumour periphery are evaluated with regard to the CLR status (*Photo 1*). The following are not evaluated as LAs associated with CLR:

1. LAs from mucosa-associated lymphoid tissue (i.e. located within the mucosal layer or just below the muscularis mucosae).
2. LAs judged to be part of small lymph nodes rather than LAs associated with CLR on the basis of the existence of circumferential connective tissue around LAs.
3. Non-nodular LAs, including irregularly shaped as well as long and narrow ones.

Small LAs are regarded as those in which the number of lymphocytes appeared to increase in comparison to those in the background but which had no definite 'aggregation', so a cut-off of 300 micro meter or larger in size is used. The largest LA in each patient is identified and its maximum diameter is determined with a calibrated ocular reticule. The outline of LAs for measurement is determined under low-power magnification.

Photos:

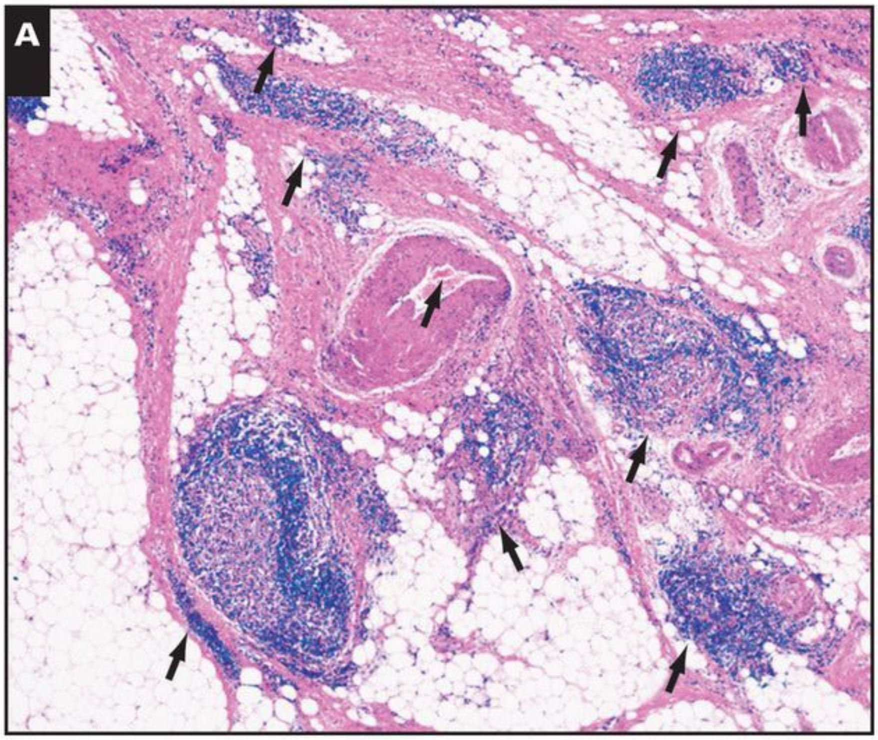

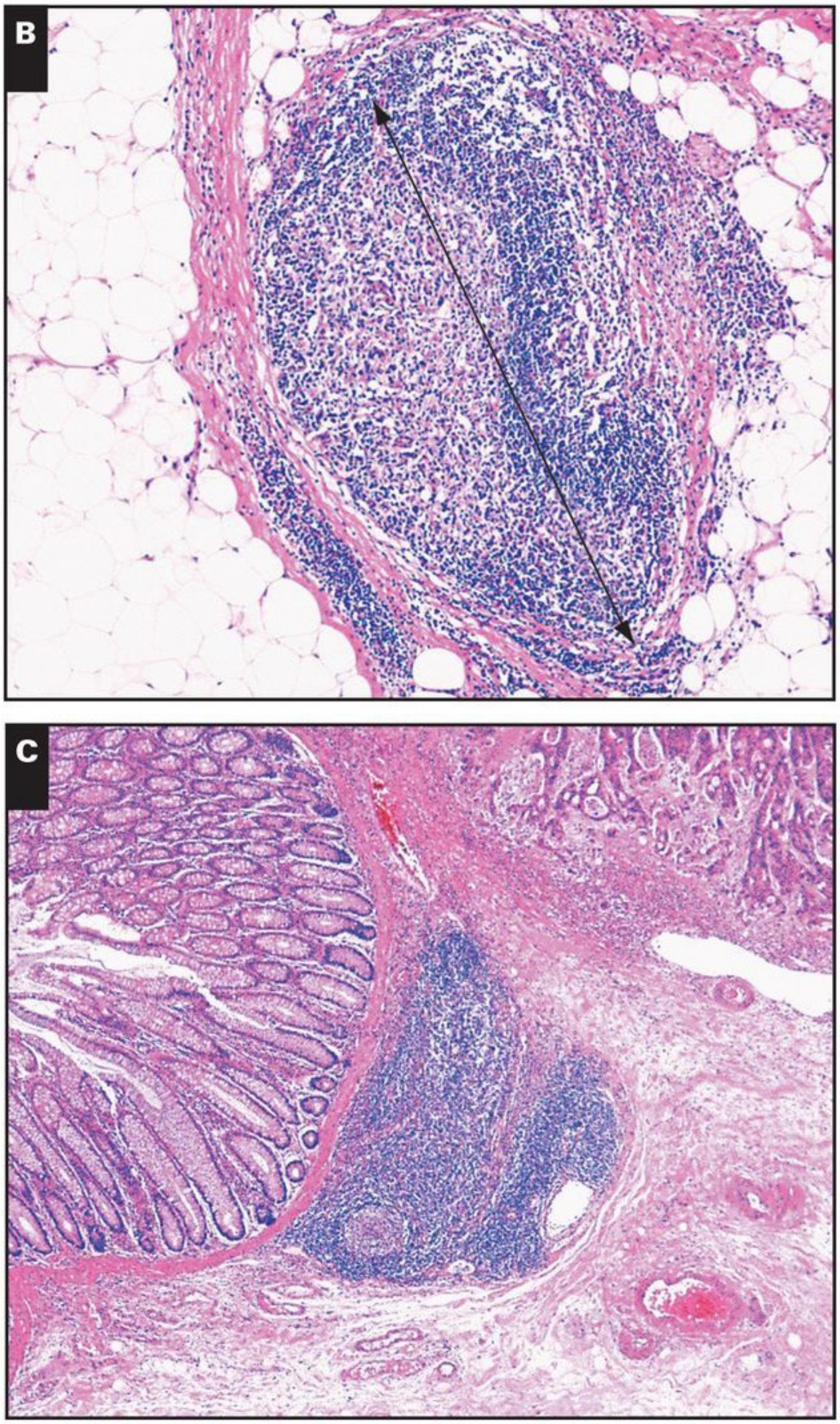

A: Multiple Crohn's-like lymphoid reactions (CLRs) of different sizes. B: Example of a large CLR. C: Lymphoid aggregates (LAs) from mucosa-associated lymphoid tissue located within the mucosal layer or just below the muscularis mucosae were not evaluated as LAs associated with CLR. (12)

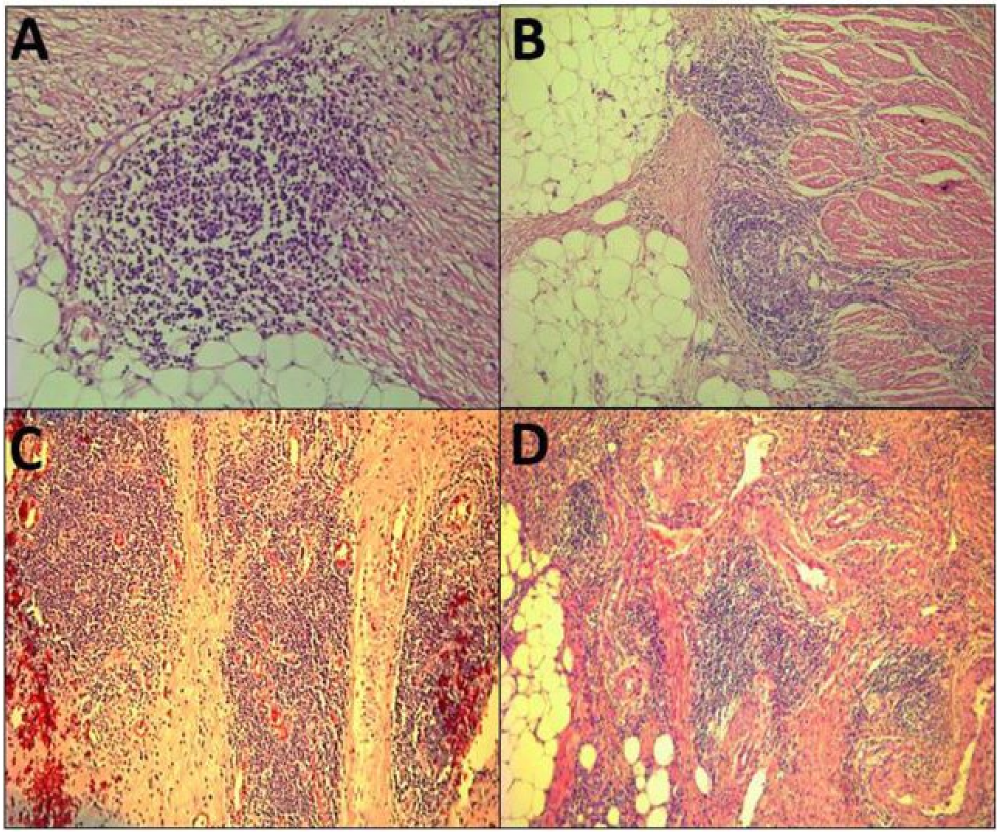

A-D: Variable pathologic appearances of the Crohn's-like lymphoid reaction. (13)

Tumour infiltrating lymphocytes (TIL)

Definition:
Tumour infiltrating lymphocytes (TIL) are determined as a percentage of mononuclear inflammatory cells over total intratumoural stromal area and counted in 5 HPF (total magnification, ×200–400) in the invasive front or areas surrounding the deposits, except for tumour areas with crush artifacts, necrosis or regressive hyalinization (14).

Examination:
See flowchart (figure 3) below.
The tumour area at low magnification is selected and the percentage of the area that is filled with mononuclear cells in the stromal area around the tumour border is assessed at high magnification (×200). Mononuclear cells are defined, including the lymphocytes in the stromal area, as TILs. Granulocytes and other polymorphonuclear leukocytes are excluded (14). The recommendation of the International TILs Working Group for evaluating the density is used (15).

Photos:

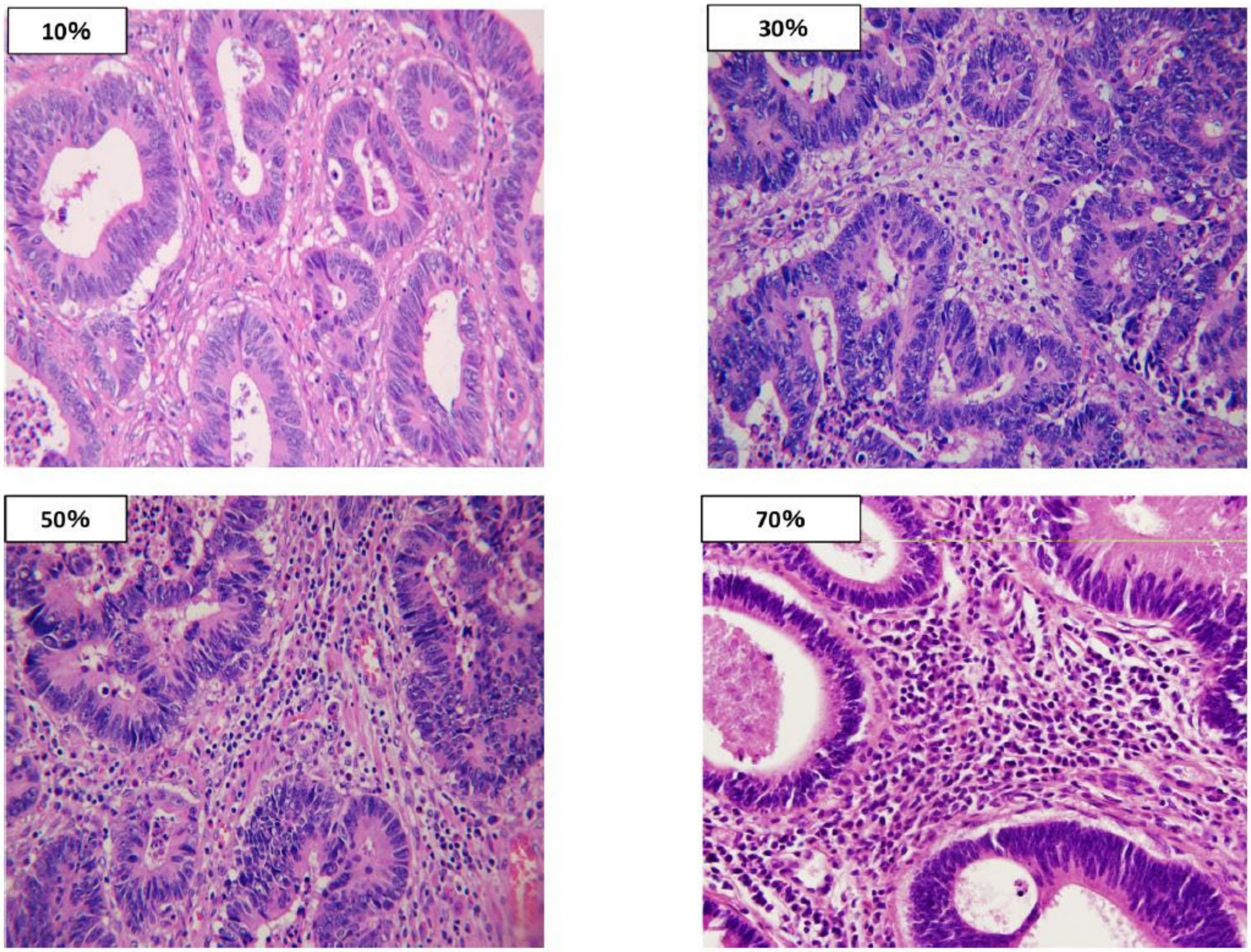

Examples of the evaluation of tumour infiltrating lymphocytes (TIL). The mean percentage of the area occupied by TILs in the intratumoural stroma is reported in each section. (14)

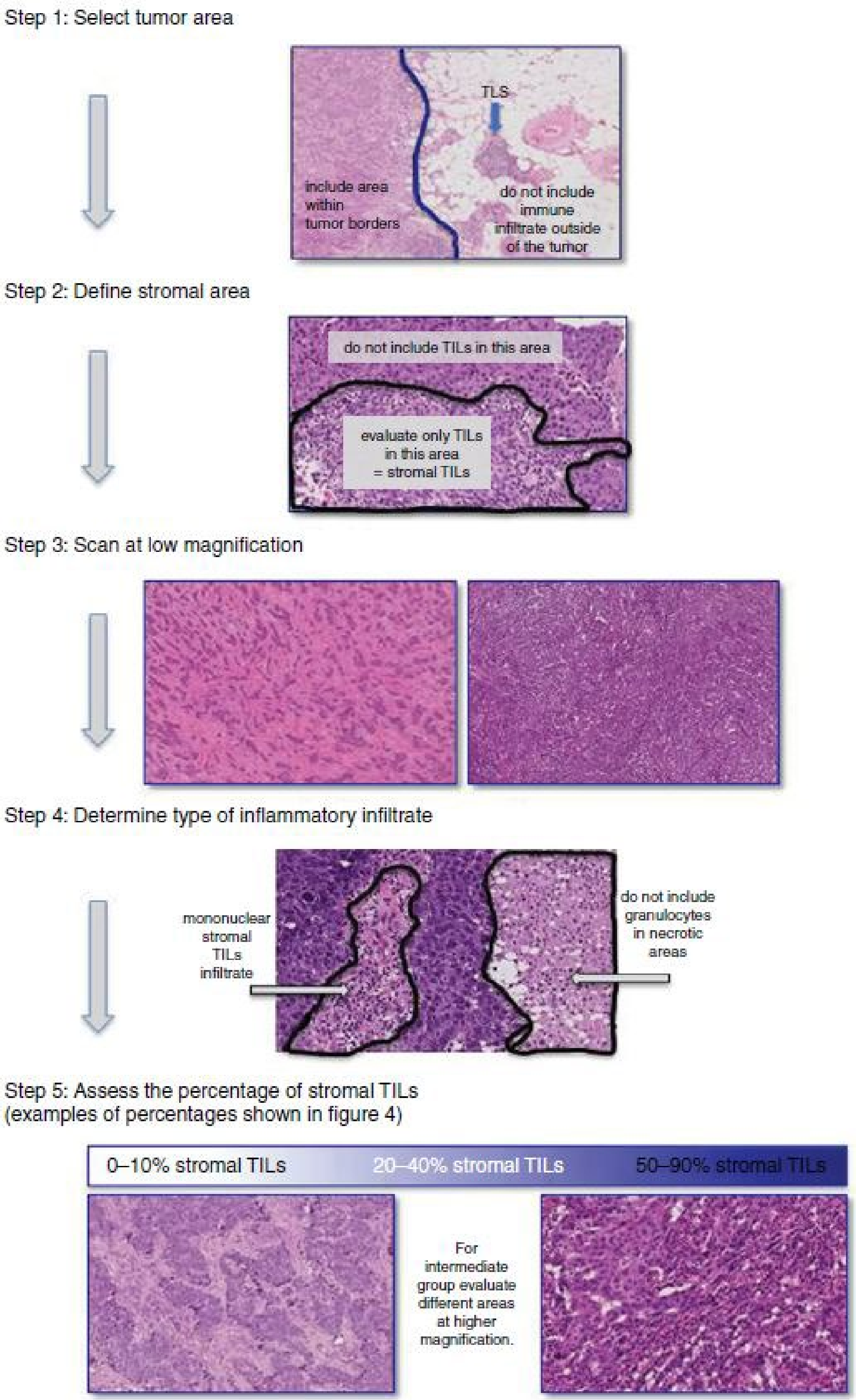

Standardized approach for TILs evaluation in breast cancer. (15)

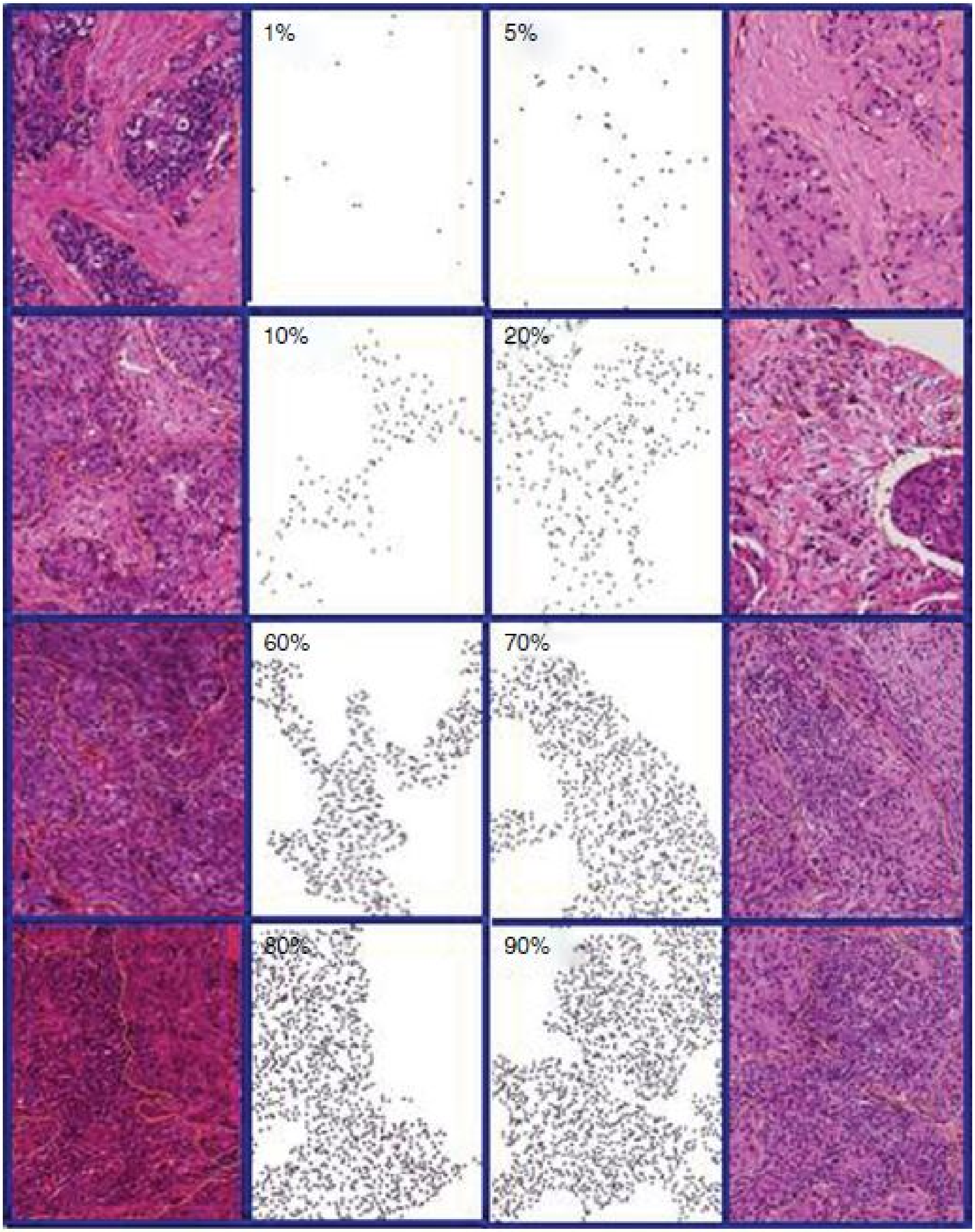

Different percentages of TILs in breast cancer (15).

References

1. Weiser MR. AJCC 8th Edition: Colorectal Cancer. Ann Surg Oncol. 2018;25(6):1454-5.
2. Frankel WL, Jin M. Serosal surfaces, mucin pools, and deposits, oh my: challenges in staging colorectal carcinoma. Mod Pathol. 2015;28 Suppl 1:S95-108.
3. Compton CC, Fielding LP, Burgart LJ, Conley B, Cooper HS, Hamilton SR, et al. Prognostic factors in colorectal cancer. College of American Pathologists Consensus Statement 1999. Arch Pathol Lab Med. 2000;124(7):979-94.
4. Betge J, Langner C. Vascular invasion, perineural invasion, and tumour budding: predictors of outcome in colorectal cancer. Acta Gastroenterol Belg. 2011;74(4):516-29.
5. Leijssen LGJ, Dinaux AM, Amri R, Taylor MS, Deshpande V, Bordeianou LG, et al. Impact of intramural and extramural vascular invasion on stage II-III colon cancer outcomes. J Surg Oncol. 2019;119(6):749-57.
6. Liebig C, Ayala G, Wilks JA, Berger DH, Albo D. Perineural invasion in cancer: a review of the literature. Cancer. 2009;115(15):3379-91.
7. Lugli A, Kirsch R, Ajioka Y, Bosman F, Cathomas G, Dawson H, et al. Recommendations for reporting tumor budding in colorectal cancer based on the International Tumor Budding Consensus Conference (ITBCC) 2016. Mod Pathol. 2017;30(9):1299-311.
8. Jass JR, Love SB, Northover JM. A new prognostic classification of rectal cancer. Lancet. 1987;1(8545):1303-6.
9. Wu JB, Sarmiento AL, Fiset PO, Lazaris A, Metrakos P, Petrillo S, et al. Histologic features and genomic alterations of primary colorectal adenocarcinoma predict growth patterns of liver metastasis. World J Gastroenterol. 2019;25(26):3408-25.
10. Ueno H, Ishiguro M, Nakatani E, Ishikawa T, Uetake H, Murotani K, et al. Prognostic value of desmoplastic reaction characterisation in stage II colon cancer: prospective validation in a Phase 3 study (SACURA Trial). Br J Cancer. 2021;124(6):1088-97.
11. Ueno H, Jones AM, Wilkinson KH, Jass JR, Talbot IC. Histological categorisation of fibrotic cancer stroma in advanced rectal cancer. Gut. 2004;53(4):581-6.
12. Ueno H, Hashiguchi Y, Shimazaki H, Shinto E, Kajiwara Y, Nakanishi K, et al. Objective criteria for crohn-like lymphoid reaction in colorectal cancer. Am J Clin Pathol. 2013;139(4):434-41.
13. Maoz A, Dennis M, Greenson JK. The Crohn's-Like Lymphoid Reaction to Colorectal Cancer-Tertiary Lymphoid Structures With Immunologic and Potentially Therapeutic Relevance in Colorectal Cancer. Front Immunol. 2019;10:1884.
14. Iseki Y, Shibutani M, Maeda K, Nagahara H, Fukuoka T, Matsutani S, et al. A new method for evaluating tumor-infiltrating lymphocytes (TILs) in colorectal cancer using hematoxylin and eosin (H-E)-stained tumor sections. PLoS One. 2018;13(4):e0192744.
15. Salgado R, Denkert C, Demaria S, Sirtaine N, Klauschen F, Pruneri G, et al. The evaluation of tumor-infiltrating lymphocytes (TILs) in breast cancer: recommendations by an International TILs Working Group 2014. Ann Oncol. 2015;26(2):259-71.
