# Supplementary material for: The relationship between primary colorectal cancer histology and the histopathological growth patterns of corresponding liver metastases
Source: BMC Cancer. 2022 Aug 22;22:911. doi: 10.1186/s12885-022-09994-3 (PMC9394040; doi:10.1186/s12885-022-09994-3)
Supplement: Supplementary file 2 — Additional file 2. Supplementary File 2. Uni- and multivariable Cox regression analysis for overall survival. [file 12885_2022_9994_MOESM2_ESM.pdf]

Supplementary File 2. Uni- and multivariable Cox regression analysis for overall survival

	Univariable		Multivariable (n=182)	
	HR [95%CI]	p-value	HR [95%CI]	p-value
CRLM characteristics				
Age at CRLM resection (cont.) - *10 years*	1.25 [1.05-1.51]	0.01	1.33 [1.10-1.62]	<0.01
Disease-free interval* (cont.) - *months*	1.00 [0.99-1.01]	0.56	-	-
Number of CRLM (cont.)	1.10 [1.00-1.21]	0.04	1.04 [0.94-1.15]	0.49
Diameter of largest CRLM (cont.) - *cm*	1.04 [0.96-1.13]	0.31	-	-
Preoperative CEA (cont.) - *100 µg/L*	1.02 [0.92-1.13]	0.73	-	-
Extrahepatic disease - *yes vs no*	1.70 [0.95-3.04]	0.07	1.61 [0.88-2.96]	0.12
Resection margin - *R1 vs R0*	0.95 [0.49-1.81]	0.87	-	-
HGP - *non-desmoplastic vs desmoplastic*	2.28 [1.31-3.99]	<0.01	1.97 [1.10-3.53]	0.02
Classical markers				
Right-sided tumour - *yes vs no*	1.86 [1.27-2.73]	<0.01	1.86 [1.23-2.83]	<0.01
Differentiation grade - *poor vs well/moderate*	2.75 [1.20-6.30]	0.02	1.49 [0.60-3.70]	0.39
pT4-stage - *yes vs no*	1.75 [1.10-2.78]	0.02	1.68 [0.94-2.98]	0.08
Positive lymph nodes - *yes vs no*	1.72 [1.19-2.49]	<0.01	1.47 [0.96-2.23]	0.07
Tumour deposits - *yes vs no*	1.37 [0.88-2.11]	0.16	1.34 [0.76-2.35]	0.31
Invasion markers				
(lympho-)vascular invasion - *yes vs no*	1.45 [1.01-2.08]	0.04	1.13 [0.75-1.71]	0.55
Extramural vascular invasion - *yes vs no*	1.33 [0.93-1.92]	0.12	0.97 [0.62-1.50]	0.88
Perineural invasion - *yes vs no*	1.35 [0.90-2.02]	0.15	1.01 [0.61-1.67]	0.97
Tumour interface markers				
Peritumoural budding - *Grade II/III vs I*	1.21 [0.77-1.91]	0.41	-	-
CRC growth pattern - *Infiltrative vs expanding*	1.39 [0.97-1.98]	0.07	1.03 [0.68-1.57]	0.88
Non-mature stroma - *yes vs no*	1.06 [0.72-1.57]	0.75	-	-
Immunological markers				
Crohn's-like lymphoid reaction - *no vs yes*	1.35 [0.80-2.28]	0.26	-	-
TIL density (cont.) - *10%*	0.92 [0.75-1.12]	0.39	-	-

** Between resection of primary tumor and detection of CRLM*

Abbreviations in alphabetical order: Cont.: entered as continous variable; CEA: carcinoembryonic antigen; CI: confidence interval; CRC: colorectal cancer; CRLM: colorectal liver metastasis; HGP: histopathological grwoth pattern; HR: hazard ratio; TIL: tumour-infiltrating lymfocyte.
